# Neuroimaging evidence of microstructural alteration in Parkinson’s disease with subjective cognitive decline

**DOI:** 10.1038/s41531-026-01313-y

**Published:** 2026-03-13

**Authors:** Kaidong Chen, Ruixuan Zhang, Yi Ji, Liujia Lu, Bin He, Yao Lu, Qin Wen, Leikun Wang, Feng Wang, Li Zhang, Xiangming Fang

**Affiliations:** 1 Department of Radiology, The Affiliated Wuxi People’s Hospital of Nanjing Medical University, Wuxi, China; 2 Department of Neurology, The Affiliated Wuxi People’s Hospital of Nanjing Medical University, Wuxi, China

**Keywords:** Biomarkers, Diseases, Neurology, Neuroscience

## Abstract

Subjective cognitive decline (SCD) may represent a special cognitive stage distinct from mild cognitive impairment (MCI) in Parkinson’s disease (PD), but its microstructural correlates are underexplored. We aimed to investigate microstructural alterations in PD-SCD compared to PD with normal cognition (PD-NC) and PD-MCI, and to explore their association with cognitive status. 68 PD patients (PD-NC: *n* = 23; PD-SCD: *n* = 20; PD-MCI: *n* = 25) and 27 healthy controls (HC) underwent multimodal MRI. Analyses included tract-based spatial statistics (TBSS), calculation of the peak width of skeletonized mean diffusivity (PSMD), and hippocampal subfield segmentation. PSMD showed significant differences across subgroups and negatively correlated with MoCA scores, suggesting its utility as a metric that differentiated cognitive stage. TBSS revealed reduced fractional anisotropy (FA) in multiple tracts in PD-MCI, while PD-SCD exhibited reduced FA only in FMajor relative to HC. Hippocampal subfield segmentation revealed atrophy in subregions such as CA1 and HATA in PD-SCD and PD-MCI compared to PD-NC. This study provides evidence that microstructural alterations are already detectable at the PD-SCD stage. PSMD emerges as a sensitive cross-sectional biomarker of PD cognitive staging. These findings highlight the potential role of white‑matter changes in PD‑related cognitive complaints.

## Introduction

Mild cognitive impairment (MCI) represents one of the most prevalent non-motor symptoms in Parkinson’s disease (PD), occurring throughout its progression^1–4^. Approximately 30% of PD patients develop MCI, representing a threefold higher risk than cognitively healthy older adults^3,5^. Cognitive decline in PD is typically gradual but can deteriorate rapidly in some cases, impairing quality of life and treatment efficacy^1^. However, identifying PD patients who may progress to MCI remains challenging, highlighting the need for reliable biomarkers associated with cognitive decline and to distinguish high-risk patients for early intervention.

Subjective cognitive decline (SCD), defined as self-reported cognitive worsening despite normal performance on objective neuropsychological assessments, is a recognized risk factor for future cognitive deterioration^6–8^. Currently, this concept has been widely applied in Alzheimer’s disease (AD) prevention and management, serving as one of the screening tools for identifying AD prodromal stages^7^. Similarly, longitudinal PD studies also show that many patients developing MCI or dementia experience prior SCD, suggesting SCD may also be a risk factor for objective cognitive decline in PD^9–12^. A review has indicated that SCD may represent a stage within the cognitive disease spectrum of PD^13^. However, research on SCD in PD remains preliminary that there is currently no consensus on the research and clinical criteria for SCD in PD^13,14^. Therefore, further research is needed to investigate PD patients in the SCD stage and identify their neuroimaging biomarker, which may facilitate the identification and mechanistic understanding of cognitive impairment and elucidate the underlying neural mechanisms of PD-related cognitive decline.

Neuroimaging provides non-invasive biomarkers of cerebral microstructure and white matter integrity, widely used in PD-MCI research^15,16^. Diffusion Tensor Imaging (DTI) studies consistently show widespread white matter damage in PD-MCI, including reduced fractional anisotropy (FA) and increased mean diffusivity (MD)^17–19^. However, the novel Peak Width of Skeletonized Mean Diffusivity (PSMD) metric, which quantifies global white matter injury, has not yet been applied in PD-MCI research^20,21^. Furthermore, hippocampal atrophy is also well-documented in PD-MCI^22^, with subfield volume reductions (e.g., cornu ammonis [CA] 1, CA3) linked to cognitive decline^23^. Yet, it remains unclear whether these neuroimaging markers are altered at the PD-SCD stage, despite the potential mechanistic insights they offer into early cognitive impairment in PD.

In summary, we hypothesized that microstructural alterations, including white matter damage and hippocampal microstructural alterations, may already be present during the SCD stage of PD. Therefore, this study would recruit PD patients stratified into three groups: PD without cognitive impairment (PD-NC), PD with SCD (PD-SCD), and PD with MCI (PD-MCI), along with age- and sex-matched healthy control (HC). Then, we investigated neuroimaging signatures of all participants, particularly white matter integrity (quantified via Tract-based Spatial Statistics [TBSS] and PSMD analysis of DTI images) and hippocampal subfield volumes (measured through hippocampal subfield segmentation of 3D-T1 images). It should be noted that the PD-SCD group in this study was operationally defined based on self-reported cognitive complaints in the presence of normal objective cognitive performance, rather than representing a standardized or biologically validated SCD phenotype, as often used in AD research. Consequently, the primary aim of this study is to explore the cross-sectional neuroimaging correlates associated with this clinically defined ‘cognitive complaint’ stage in PD, thereby providing preliminary evidence for its potential neurobiological underpinnings.

## Results

### Population and Participant Characteristics

The study included 68 PD patients and 27 HCs. PD patients were categorized into three groups: PD-MCI (*n* = 25), PD-SCD (*n* = 20), and PD-NC (*n* = 23). No significant differences were observed in gender, age, education, Hoehn & Yahr (H&Y) stage, levodopa equivalent daily dose (LEDD), Unified Parkinson’s Disease Rating Scale (UPDRS)-II/III scores, or The Non-Motor Symptoms Scale for Parkinson’s disease (NMSS) among groups (all *p* > 0.05). PD-MCI and PD-SCD groups exhibited higher Hamilton Depression Rating Scale (HAM-D) scores than HC (*p* = 0.006 and *p* = 0.017, respectively). All PD subgroups showed elevated Hamilton Anxiety Rating Scale (HAM-A) scores (*p* < 0.01). However, no statistically significant differences in HAM-D or HAM-A scores were observed among the PD subgroups (all *p* > 0.05). All results above were corrected for multiple comparisons using Bonferroni method. As presented in Table 1 and Supplementary Fig. S1.

**Table 1:** Intergroup comparisons of demographic features and clinical profiles using ANOVA

	PD-MCI (*n* = 25)	PD-SCD (*n* = 20)	PD-NC (*n* = 23)	HC (*n* = 27)	*p* value	η²
Gender (M/F)	15/10	11/9	14/9	12/15	0.618	0.019
Age (years)	65.08 ± 7.01	65.95 ± 6.55	61.83 ± 10.94	62.96 ± 8.59	0.346	0.036
Education (years)	9.16 ± 3.51	10.03 ± 2.59	10.50 ± 2.62	9.63 ± 4.07	0.552	0.023
Duration (years)	4.38 ± 3.24	3.88 ± 2.85	3.85 ± 2.92	NA	0.792	0.007
H&Y	2.06 ± 0.78	1.93 ± 0.77	2.11 ± 0.66	NA	0.704	0.011
LEDD (mg)	471.00 ± 275.37	378.75 ± 223.06	370.11 ± 145.66	NA	0.231	0.044
UPDRS-Ⅰ, Item 1	2.32 ± 1.11^ab^	1.25 ± 0.64^b^	0 ± 0	NA	< 0.001***	0.634
UPDRS-Ⅱ	10.20 ± 5.45	10.35 ± 4.52	8.87 ± 3.73	NA	0.504	0.021
UPDRS-Ⅲ	23.64 ± 12.88	20.65 ± 10.33	22.48 ± 9.74	NA	0.672	0.012
FAB	16.56 ± 1.73^b^	17.30 ± 0.92	17.52 ± 0.73	NA	0.024*	0.108
NMSS	48.08 ± 46.36	48.15 ± 28.99	38.91 ± 31.62	NA	0.629	0.014
NMSS item16	2.20 ± 3.45^b^	1.40 ± 2.62	0 ± 0	NA	0.014*	0.124
NMSS item17	3.20 ± 2.92^b^	2.75 ± 2.79^b^	0 ± 0	NA	< 0.001***	0.284
NMSS item18	3.28 ± 3.62^b^	2.45 ± 2.48^b^	0 ± 0	NA	< 0.001***	0.240
MoCA	22.60 ± 2.00^abc^	27.15 ± 0.99^c^	27.83 ± 1.23	28.59 ± 1.22	< 0.001***	0.746
MoCA-visuospatial/executive	3.64 ± 1.19^abc^	4.55 ± 0.76	4.61 ± 0.58	4.78 ± 0.42	< 0.001***	0.258
MoCA-memory	6.80 ± 1.04^abc^	8.85 ± 1.27^bc^	9.61 ± 1.12	9.96 ± 0.98	< 0.001***	0.582
MoCA-attention	7.76 ± 1.23^abc^	8.90 ± 0.31	8.83 ± 0.39	8.96 ± 0.19	< 0.001***	0.362
MoCA-language	6.32 ± 1.31^abc^	7.15 ± 0.93^c^	7.26 ± 0.81^c^	7.85 ± 0.36	< 0.001***	0.289
HAM-D	7.44 ± 6.70^c^	7.25 ± 5.04^c^	5.26 ± 4.48	2.89 ± 1.99	0.003**	0.139
HAM-A	10.68 ± 8.94^c^	9.95 ± 6.03^c^	8.65 ± 7.33^c^	2.56 ± 1.87	< 0.001***	0.214

Significance levels: *p < 0.05, **p < 0.01, ***p < 0.001.

Post hoc two-sample t-test: ^a^ significant difference compared to the PD-SCD group; ^b^ significant difference compared to the PD-NC group; ^c^ significant difference compared to the HC group.

*PD* Parkinson’s disease, *MCI* mild cognitive impairment, *SCD* subjective cognitive decline, *NC* non cognitive impairment, *HC* healthy control, *H&Y* Hoehn & Yahr scale, *LEDD* levodopa equivalent daily dose, *UPDRS* Unified Parkinson’s Disease Rating Scale, *FAB* Frontal Assessment Battery, *NMSS* The Non-Motor Symptoms Scale for Parkinson’s disease, *MoCA* Montreal Cognitive Assessment, *HAM-D* 17-item Hamilton Depression Rating Scale, *HAM-A* Hamilton Anxiety Rating Scale, *NA* not applicable.

### Cognitive assessment

Montreal Cognitive Assessment (MoCA) scores differed significantly among groups (*p* < 0.001). Post-hoc tests showed PD-MCI scored lower than all others (all *p* < 0.001), while PD-SCD scored lower than HC (*p* = 0.006) but not PD-NC (*p* = 0.056). In subdomains, PD-MCI showed impairment in visuospatial/executive and attention functions compared to all others (all *p* < 0.01), whereas no differences were found among PD-SCD, PD-NC, and HC. MoCA-memory scores were severely impaired in PD-MCI (all *p* < 0.001), with PD-SCD trending lower than PD-NC (*p* = 0.043) and HC (*p* = 0.001). In language, PD-MCI scored lower than all others (all *p* < 0.05), and HC outperformed both PD-SCD and PD-NC (*p* < 0.01). Frontal Assessment Battery (FAB) scores were lower in PD-MCI than HC (*p* = 0.028). UPDRS-I item 1 scores and NMSS (item 16, 17, 18) differed across PD subgroups (PD-MCI > PD-SCD > PD-NC, all *p* < 0.05). All reported results were Bonferroni-corrected. All details could be found in Table 1 and Supplementary Figure S1.

### Neuroimaging markers

PSMD showed significant group differences in ANCOVA after covariate adjustment: in comparisons across all four groups, the group effect was significant after adjusting for age, sex, education, and mood scores (F = 8.129, *p* < 0.001, η² = 0.221); among PD subgroups, the difference remained significant after controlling for the above covariates as well as additional LEDD (F = 6.404, *p* = 0.003, η² = 0.178). Post-hoc analyses for comparisons across four groups demonstrated that PD-MCI exhibited significantly higher PSMD than all other groups (vs. PD-SCD: *p* = 0.048, Cohen’s d [95% CI] = 0.609 [0.007, 1.211]; vs. PD-NC: *p* = 0.002, Cohen’s d [95% CI] = 0.994 [0.392, 1.596]; vs. HC: *p* < 0.001, Cohen’s d [95% CI] = 1.288 [0.688, 1.888]). Critically, PD-SCD also demonstrated elevated PSMD compared to both PD-NC (*p* = 0.035, Cohen’s d [95% CI] = 0.650 [0.034, 1.266]) and HC (*p* < 0.001, Cohen’s d [95% CI] = 1.255 [0.621, 1.889]).

ANCOVA analyses performed on the TBSS-FA data revealed that widespread white matter alterations among the groups (all *p* < 0.05). For outcomes that showed significance in both the full cohort analysis (covariates: age, sex, education, HAMD, HAMA) and the PD subgroup analysis (additional covariate: LEDD), post hoc two-sample t-tests were subsequently conducted across the four groups. Compared to PD-NC, PD-MCI showed significantly reduced FA in bilateral anterior thalamic radiation (ATR) (left: *p* = 0.013; right: *p* = 0.012), bilateral inferior fronto-occipital fasciculus (IFOF) (left: *p* = 0.004; right: *p* = 0.046), bilateral superior longitudinal fasciculus (SLF) (left: *p* = 0.045; right: *p* = 0.03), left uncinate fasciculus (UNF) (*p* = 0.012), forceps major (FMajor) (*p* = 0.02), and forceps minor (FMinor) (*p* = 0.03). PD-MCI also exhibited lower FA than HC in these tracts except right ATR and left UNF (all *p* < 0.05). Notably, PD-SCD had reduced FA only in FMajor relative to HC (*p* = 0.048), while no differences were found between PD-NC and HC.

Volume of whole hippocampus showed no significance, but some of hippocampal subfield volumes differed significantly among the groups (all *p* < 0.05). As done previously, post hoc two-sample t-tests were performed on hippocampal subfield volumes that showed significant differences in both the ANCOVA across all four groups and the ANCOVA among PD subgroups, including CA1-head, granule cell and molecular layer of the dentate gyrus (GMD)-head, and hippocampal-amygdaloid transition area (HATA). Post-hoc tests showed that compared to PD-NC, PD-MCI and PD-SCD both had reduced volumes in CA1-head (PD-MCI vs. PD-NC: *p* = 0.018; PD-SCD vs. PD-NC: *p* = 0.047), GMD-head (PD-MCI vs. PD-NC: *p* = 0.036), and HATA. (PD-MCI vs. PD-NC: *p* = 0.005; PD-SCD vs. PD-NC: *p* = 0.037). PD-MCI and PD-SCD also exhibited volume reductions of CA1-head (PD-MCI vs. HC: *p* = 0.017; PD-SCD vs. HC: *p* = 0.028) and HATA (PD-MCI vs. HC: *p* = 0.023; PD-SCD vs. HC: *p* = 0.044) compared to HC.

**Fig. 1:**
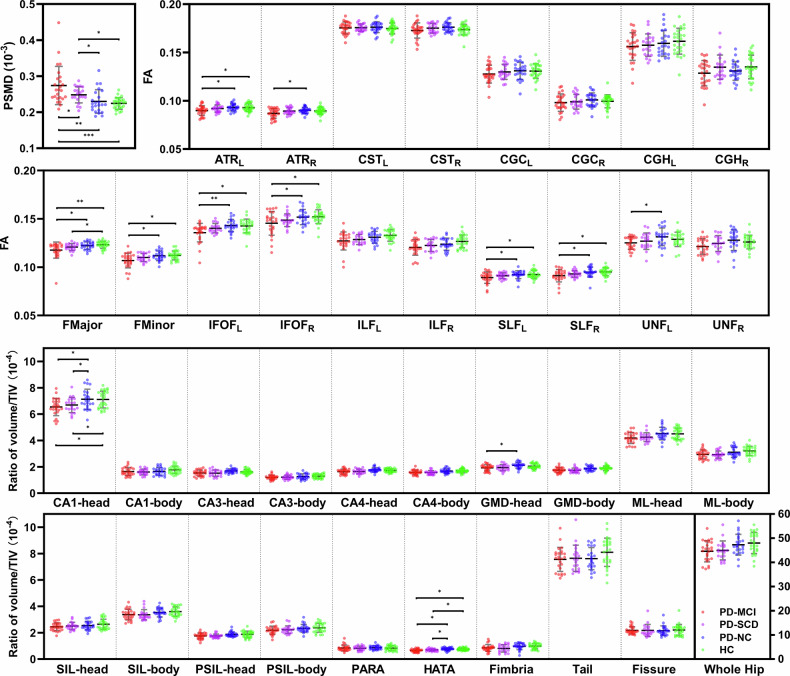
Comparison of neuroimaging biomarkers, including PSMD, TBSS-FA, and hippocampal subfield volumes, among PD-MCI (*N* = 25), PD-SCD (*N* = 20), PD-NC (*N* = 23), and HC (*N* = 27) with ANCOVA. ANCOVA analyses across all four groups were performed adjusted for age, sex, education, HAMD, and HAMA; ANCOVA analyses among PD subgroups were performed adjusted for above covariates and additional LEDD to minimize the influence of medication factors on the results. Post hoc two-sample t-test was conducted among the four groups for the outcomes that demonstrated significance in both the comparison among PD subgroups and across all four groups. Error bars represent the standard error of the mean. Asterisks indicate statistically significant group differences (*: *p* < 0.05, **: *p* < 0.01,***: *p* < 0.001). PD Parkinson’s disease, MCI mild cognitive impairment, SCD subjective cognitive decline, NC non cognitive impairment, HC healthy control, PSMD peak width of skeletonized mean diffusivity, TBSS tract-based spatial statistics, FA fractional anisotropy, ATR anterior thalamic radiation, CST corticospinal tract, CGC cingulum-cingulate gyrus, CGH cingulum-hippocampus, FMajor forceps major, FMinor forceps minor, IFOF inferior fronto-occipital fasciculus, ILF inferior longitudinal fasciculus, SLF superior longitudinal fasciculus, UNF uncinate fasciculus, _L_ left hemisphere, _R_ right hemisphere, CA cornu ammonis, GMD granule cell and molecular layer of the dentate gyrus, ML molecular layer, SIL subiculum, PSIL presubiculum, PARA parasubiculum, HATA hippocampal-amygdaloid transition area.

Bonferroni correction was applied to all above results. All details are demonstrated in Fig. 1 and Table 2.

**Table 2:** Comparative analysis of neuroimaging biomarkers between groups using ANCOVA

	PD-MCI (*n* = 25)	PD-SCD (*n* = 20)	PD-NC (*n* = 23)	HC (*n* = 27)	across all groups			among PD subgroups		
					*F*	*p*	η²	*F*	*p*	η²
PSMD^d^	0.274 ± 0.053^abc^	0.248 ± 0.022^bc^	0.230 ± 0.032	0.224 ± 0.016	8.129	0.001***	0.221	6.404	0.003**	0.178
TBSS-FA										
ATR_L_^d^	0.090 ± 0.005^bc^	0.092 ± 0.003	0.093 ± 0.003	0.093 ± 0.004	2.706	0.05*	0.086	3.793	0.028*	0.114
ATR_R_^d^	0.087 ± 0.005^bc^	0.089 ± 0.003	0.090 ± 0.003	0.089 ± 0.004	2.817	0.044*	0.089	5.304	0.008**	0.152
CST_L_	0.175 ± 0.006	0.175 ± 0.004	0.176 ± 0.006	0.175 ± 0.006	0.159	0.924	0.006	0.194	0.824	0.007
CST_R_	0.173 ± 0.008	0.175 ± 0.005	0.176 ± 0.005	0.174 ± 0.005	1.685	0.176	0.056	2.788	0.07	0.086
CGC_L_	0.128 ± 0.009	0.130 ± 0.008	0.131 ± 0.009	0.131 ± 0.008	0.813	0.49	0.028	0.779	0.464	0.026
CGC_R_	0.098 ± 0.009	0.099 ± 0.008	0.101 ± 0.007	0.099 ± 0.007	0.277	0.842	0.01	0.496	0.611	0.017
CGH_L_	0.156 ± 0.014	0.157 ± 0.012	0.159 ± 0.013	0.162 ± 0.013	0.443	0.723	0.015	0.094	0.911	0.003
CGH_R_	0.129 ± 0.013	0.135 ± 0.013	0.131 ± 0.010	0.135 ± 0.012	1.318	0.274	0.044	1.466	0.239	0.047
FMajor^d^	0.118 ± 0.008^bc^	0.121 ± 0.004^c^	0.122 ± 0.004	0.121 ± 0.004	4.394	0.006**	0.129	3.302	0.043*	0.095
FMinor^d^	0.107 ± 0.008^bc^	0.110 ± 0.004	0.112 ± 0.005	0.112 ± 0.005	3.111	0.031*	0.098	3.841	0.027*	0.115
IFOF_L_^d^	0.136 ± 0.010^bc^	0.140 ± 0.005	0.143 ± 0.006	0.143 ± 0.007	4.01	0.009**	0.123	4.756	0.012*	0.139
IFOF_R_	0.145 ± 0.012	0.149 ± 0.007	0.152 ± 0.008	0.152 ± 0.007	3.207	0.027*	0.096	3.148	0.05*	0.095
ILF_L_	0.127 ± 0.009	0.129 ± 0.005	0.131 ± 0.006	0.133 ± 0.006	1.811	0.151	0.059	1.037	0.361	0.034
ILF_R_	0.120 ± 0.008	0.122 ± 0.006	0.124 ± 0.006	0.127 ± 0.007	1.67	0.179	0.055	1.528	0.225	0.049
SLF_L_^d^	0.089 ± 0.006^bc^	0.091 ± 0.003	0.092 ± 0.004	0.092 ± 0.004	2.755	0.047*	0.088	3.545	0.035*	0.107
SLF_R_^d^	0.091 ± 0.006^bc^	0.093 ± 0.003	0.095 ± 0.005	0.095 ± 0.004	4.005	0.01**	0.123	3.699	0.031*	0.111
UNF_L_^d^	0.125 ± 0.007^b^	0.127 ± 0.009	0.131 ± 0.009	0.129 ± 0.007	3.165	0.029*	0.099	4.552	0.015*	0.134
UNF_R_	0.121 ± 0.008	0.125 ± 0.008	0.128 ± 0.011	0.126 ± 0.007	2.624	0.056	0.084	3.871	0.026*	0.116
Hippocampal Volume										
Whole hippocampus	44.582 ± 4.347	44.917 ± 3.907	47.287 ± 4.299	47.998 ± 4.398	2.444	0.07	0.079	1.242	0.296	0.04
CA1-head^d^	6.540 ± 0.654^bc^	6.696 ± 0.577^bc^	7.126 ± 0.769	7.108 ± 0.641	3.644	0.016*	0.113	4.756	0.012*	0.128
CA1-body	1.640 ± 0.306	1.609 ± 0.224	1.658 ± 0.293	1.771 ± 0.283	1.273	0.289	0.043	0.209	0.812	0.007
CA3-head	1.531 ± 0.227	1.521 ± 0.244	1.691 ± 0.159	1.625 ± 0.176	2.459	0.068	0.079	3.041	0.055	0.093
CA3-body	1.201 ± 0.169	1.213 ± 0.159	1.266 ± 0.210	1.291 ± 0.159	0.38	0.768	0.013	0.364	0.696	0.012
CA4-head	1.639 ± 0.193	1.647 ± 0.183	1.658 ± 0.293	1.770 ± 0.167	2.345	0.079	0.076	2.436	0.096	0.076
CA4-body	1.582 ± 0.175	1.565 ± 0.150	1.679 ± 0.173	1.692 ± 0.149	2.042	0.114	0.067	1.666	0.198	0.053
GMD-head^d^	1.922 ± 0.235^b^	1.938 ± 0.237	2.116 ± 0.220	2.047 ± 0.194	2.973	0.036*	0.094	3.558	0.034*	0.103
GMD-body	1.747 ± 0.197	1.737 ± 0.170	1.874 ± 0.172	1.891 ± 0.171	2.698	0.051	0.086	2.114	0.13	0.067
ML-head	4.187 ± 0.422	4.252 ± 0.335	4.520 ± 0.474	4.501 ± 0.436	2.887	0.04*	0.092	2.309	0.108	0.073
ML-body	2.942 ± 0.341	2.930 ± 0.273	3.104 ± 0.378	3.207 ± 0.357	1.784	0.156	0.059	0.83	0.441	0.027
SIL-head	2.441 ± 0.299	2.507 ± 0.282	2.541 ± 0.320	2.639 ± 0.386	1.689	0.175	0.056	0.573	0.567	0.019
SIL-body	3.372 ± 0.404	3.360 ± 0.388	3.533 ± 0.342	3.600 ± 0.357	1.359	0.261	0.045	0.665	0.518	0.022
PSIL-head	1.753 ± 0.222	1.752 ± 0.146	1.880 ± 0.186	1.873 ± 0.246	1.935	0.13	0.063	1.767	0.18	0.057
PSIL-body	2.186 ± 0.310	2.226 ± 0.295	2.330 ± 0.306	2.372 ± 0.346	1.159	0.33	0.039	0.397	0.674	0.013
PARA	0.840 ± 0.231	0.819 ± 0.167	0.866 ± 0.154	0.827 ± 0.192	0.401	0.753	0.014	0.193	0.825	0.007
HATA^d^	0.649 ± 0.116^bc^	0.689 ± 0.097^bc^	0.760 ± 0.115	0.750 ± 0.099	3.587	0.017*	0.111	3.873	0.026*	0.116
Fimbria	0.849 ± 0.255	0.810 ± 0.285	0.960 ± 0.243	0.982 ± 0.207	1.71	0.171	0.056	0.662	0.519	0.022
Tail	7.559 ± 0.914	7.645 ± 0.997	7.615 ± 0.839	8.098 ± 1.075	1.787	0.156	0.059	0.216	0.806	0.007
Fissure	2.178 ± 0.278	2.177 ± 0.455	2.151 ± 0.368	2.206 ± 0.427	0.035	0.991	0.001	0.005	0.995	0.001

ANCOVA analyses across all four groups were performed adjusted for age, sex, education, HAMD, and HAMA; ANCOVA analyses among PD subgroups were performed adjusted for not only above covariates but also LEDD to minimize the influence of medication factors on the results.

*PD* Parkinson’s disease, *MCI* mild cognitive impairment, *SCD* subjective cognitive decline, *NC* non cognitive impairment, *HC* healthy control, *PSMD* peak width of skeletonized mean diffusivity, *TBSS* tract-based spatial statistics, *FA* fractional anisotropy, *ATR* anterior thalamic radiation, *CST* corticospinal tract, *CGC* cingulum-cingulate gyrus, *CGH* cingulum-hippocampus, *FMajor* forceps major, *FMinor* forceps minor, *IFOF* inferior fronto-occipital fasciculus, *ILF* inferior longitudinal fasciculus, *SLF* superior longitudinal fasciculus, *UNF* uncinate fasciculus, _L_ left hemisphere, _R_ right hemisphere, *CA* cornu ammonis, *GMD* granule cell and molecular layer of the dentate gyrus, *ML* molecular layer, *SIL* subiculum, *PSIL* presubiculum, *PARA* parasubiculum, *HATA* hippocampal-amygdaloid transition area.

Significance levels: *p < 0.05, **p < 0.01, ***p < 0.001, corrected by Bonferroni.

^d^ Post hoc two-sample t-test was conducted among the four groups for the outcomes that demonstrated significance in both the comparison among PD subgroups and across all four groups: ^a^ significant difference compared to the PD-SCD group; ^b^ significant difference compared to the PD-NC group; ^c^ significant difference compared to the HC group.

### Correlation analysis

According to partial correlation analysis after adjusting for age, gender, education, HAM-D, HAM-A, and LEDD, PSMD negatively correlated with total MoCA (*r* = −0.401, *p* < 0.001), memory (*r* = −0.416, *p* < 0.001), and attention (*r* = −0.287, *p* = 0.023) scores, and positively with UPDRS-I (item 1) (*r* = 0.324, *p* = 0.01).

According to partial correlation analysis after controlling for covariates, FA values in multiple tracts, including bilateral ATR, IFOF, SLF, UNF, right corticospinal tract (CST), and FMinor, positively correlated with total MoCA scores (all *p* < 0.05). MoCA-memory was positively associated with FA in a broader set of tracts, consist of above tracts, positively related to MoCA and additional FMajor and right inferior longitudinal fasciculus (ILF) (all *p* < 0.05). MoCA-attention and MoCA-language subscores also correlated with specific tract FAs (MoCA-attention with right ATR; MoCA-language with right CST, left SLF, right UNF, and FMinor) (all *p* < 0.05). UPDRS-I item 1 scores negatively correlated with FA in right ATR, bilateral UNF, FMajor, and left IFOF (all *p* < 0.05).

According to partial correlation analysis controlled for covariates, HATA volume positively correlated with memory scores (*r* = 0.333, *p* = 0.008). CA3-head, CA4-head, GMD-head, and HAMA volumes negatively correlated with UPDRS-I item 1 scores (all *p* < 0.05).

**Fig. 2:**
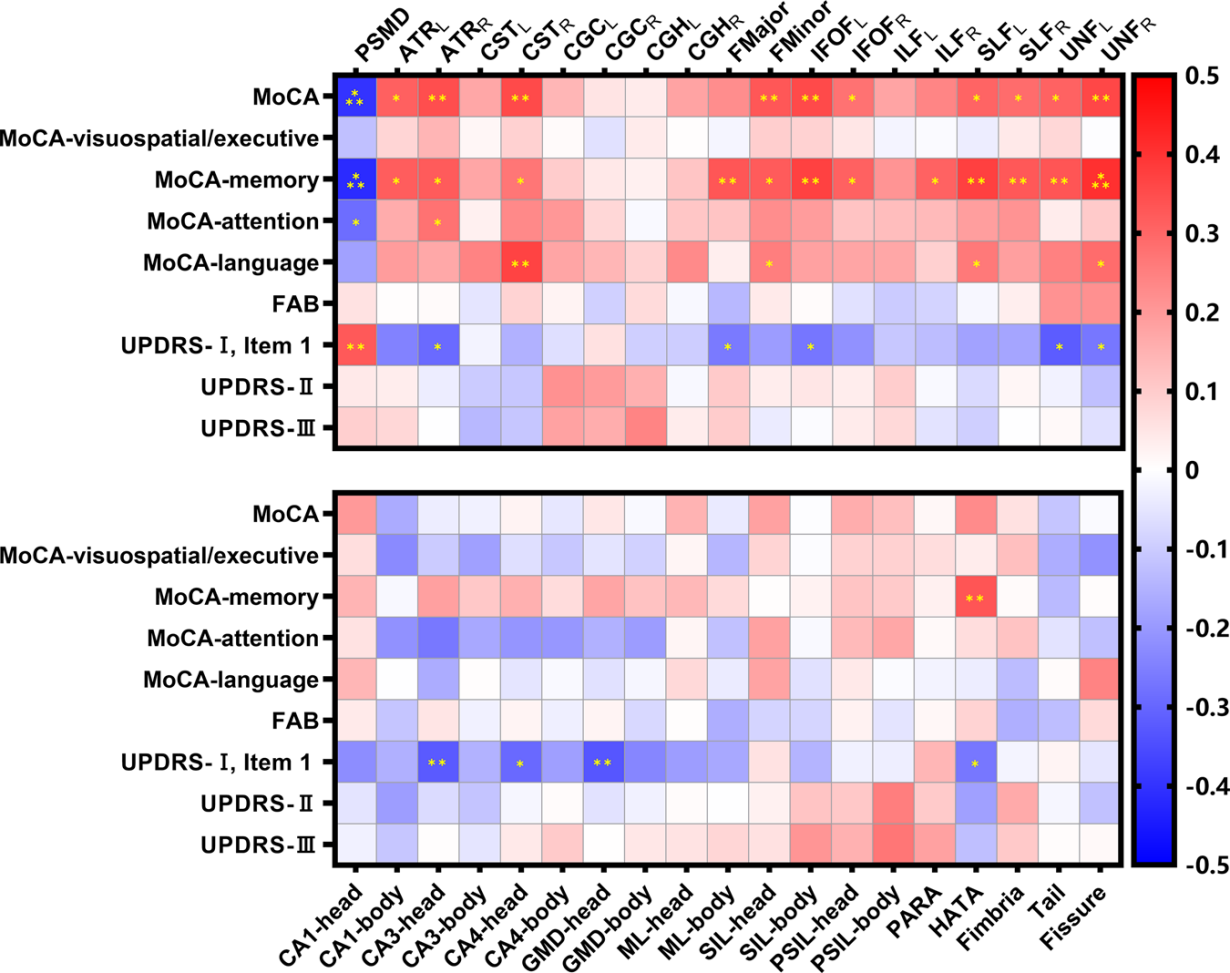
Partial correlation analysis between clinical features and neuroimaging biomarkers in PD cohort (*N* = 68), including PSMD, TBSS-FA, and hippocampal subfield volumes, after controlling for age, gender, education, HAM-D, HAM-A, and LEDD. Asterisks indicate statistically significant group differences (*: *p* < 0.05, **: *p* < 0.01,***: *p* < 0.001). PD Parkinson’s disease, LEDD levodopa equivalent daily dose, H&Y Hoehn & Yahr scale, MoCA Montreal Cognitive Assessment, FAB Frontal Assessment Battery, UPDRS Unified Parkinson’s Disease Rating Scale, HAM-D 17-item Hamilton Depression Rating Scale, HAM-A Hamilton Anxiety Rating Scale, PSMD peak width of skeletonized mean diffusivity, ATR anterior thalamic radiation, CST corticospinal tract, CGC cingulum-cingulate gyrus, CGH cingulum-hippocampus, FMajor forceps major, FMinor forceps minor, IFOF inferior fronto-occipital fasciculus, ILF inferior longitudinal fasciculus, SLF superior longitudinal fasciculus, UNF uncinate fasciculus, _L_ left hemisphere, _R_ right hemisphere, CA cornu ammonis, GMD granule cell and molecular layer of the dentate gyrus, ML molecular layer, SIL subiculum, PSIL presubiculum, PARA parasubiculum, HATA hippocampal-amygdaloid transition area.

All results of correlation analyses were corrected by Bonferroni method. As presented in Fig. 2 and Supplementary Table S1.

### Ordinal logistic regression analysis

With adjustments made for age, gender, education, HAM-D, HAM-A, and LEDD, a univariate analysis showed that PSMD alone significantly associated with cognitive staging (χ² = 23.895, *p* < 0.001), yielding a Nagelkerke pseudo-R² of 0.334, suggesting a moderate model fit. Remarkably, PSMD was a significant positive discriminator (*B* = 1.132, *p* < 0.001), with an OR of 3.102 (95% CI: 1.562–6.164) per standard deviation increase.

With adjustments made for covariates, a multivariate model including nine significant FA tracts was overall significant (χ² = 28.347, *p* = 0.013), yielding a Nagelkerke pseudo-R² of 0.384, indicating acceptable model fit. Bilateral IFOF FAs were independent discriminators (left: *B* = −1.725, *p* = 0.028, OR [95% CI] = 0.178 [0.038, 0.827]; right: *B* = −1.772, *p* = 0.011, OR [95% CI] = 0.169 [0.043, 0.667]). Collinearity diagnostics revealed that all VIF values for the variables included in the model were below 10.

Finally, after adjusting for covariates, a multivariate model incorporating three hippocampal subfield volumes demonstrated significant efficacy in differentiating among PD cognitive subgroups (χ² = 20.037, *p* = 0.018), yielding a Nagelkerke pseudo-R² of 0.287, suggesting modest model fit. HATA was identified as a significant protective factor (*B* = −0.664, *p* = 0.049, OR [95% CI] = 0.515 [0.266, 0.996]).

All ordinal logistic regression analysis results in this section were corrected by Bonferroni. Detailed results are shown in Table 3 and Table 4.

**Table 3:** Ordinal logistic regression analysis of PSMD, TBSS-FA and Hippocampal Subfield Volume for discriminating PD cognition-related subgroups adjusted for age, sex, education, LEDD, HAMD, and HAMA

Variable	*B* (Estimate)	SE	Wald	*p* value	OR [95% CI]
PSMD Model					
PSMD	1.132	0.350	10.451	< 0.001***	3.102 [1.562, 6.164]
TBSS-FA Model					
ATR_L_	0.804	0.648	1.538	0.215	2.234 [0.628, 7.967]
ATR_R_	−0.946	0.626	2.281	0.131	0.388 [0.114, 1.326]
FMajor	–0.678	0.535	1.604	0.205	0.508 [0.178, 1.449]
FMinor	0.177	0.491	0.130	0.718	1.193 [0.456, 3.126]
IFOF_L_	–1.725	0.783	4.852	0.028*	0.178 [0.038, 0.827]
IFOF_R_	−1.772	0.697	6.455	0.011*	0.169 [0.043, 0.667]
SLF_L_	–0.329	0.596	0.304	0.581	0.720 [0.224, 2.315]
SLF_R_	−0.288	0.604	0.227	0.634	0.750 [0.230, 2.453]
UNF_L_	−0.403	0.390	1.070	0.301	0.668 [0.311, 1.434]
Hippocampal Subfield Volume Model					
CA1-head	−0.500	0.576	0.755	0.385	0.607 [0.196, 1.874]
GMD-head	0.229	0.519	0.194	0.659	1.257 [0.454, 3.480]
HATA	−0.664	0.337	3.892	0.049*	0.515 [0.266, 0.996]

Significance levels: *p < 0.05, ***p < 0.001.

*PD* Parkinson’s disease, *B* regression coefficient, *SE* standard error, *OR* odds ratio (Exp[β]), *CI* confidence interval, *PSMD* peak width of skeletonized mean diffusivity, *TBSS* tract-based spatial statistics, *FA* fractional anisotropy, *ATR* anterior thalamic radiation, *FMajor* forceps major, *FMinor* forceps minor, *IFOF* inferior fronto-occipital fasciculus, *SLF* superior longitudinal fasciculus, *UNF* uncinate fasciculus, _L_ left hemisphere, _R_ right hemisphere, *CA* cornu ammonis, *GMD* granule cell and molecular layer of the dentate gyrus, *HATA* hippocampal-amygdaloid transition area.

**Table 4:** Model fit statistics for all ordinal logistic regression models with age, sex, education, LEDD, HAMD, and HAMA as covariates

Model	−2 Log Likelihood	Model χ²	df	*p* value	Nagelkerke’s pseudo R²	Parallel Lines Test (*p*)
PSMD	124.953	23.895	7	<0.001***	0.334	0.798
TBSS-FA	120.501	28.347	15	0.013*	0.384	0.821
Hippocampal Subfield Volume	128.810	20.037	9	0.018*	0.287	0.777
Null Model	148.847					

The −2 Log Likelihood for the null model is provided for each corresponding analysis. The TBSS-FA Model included 9 predictors, including the FA values of bilateral ATR, bilateral IFOF, bilateral ILF, left UNF, FMajor, and FMinor; the Hippocampal Subfield Model included 3 predictors, including the volumes of CA1-head, GMD-head, and HATA.

Significance levels: *p < 0.05, ***p < 0.001.

*df* degrees of freedom.

## Discussion

**Fig. 3:**
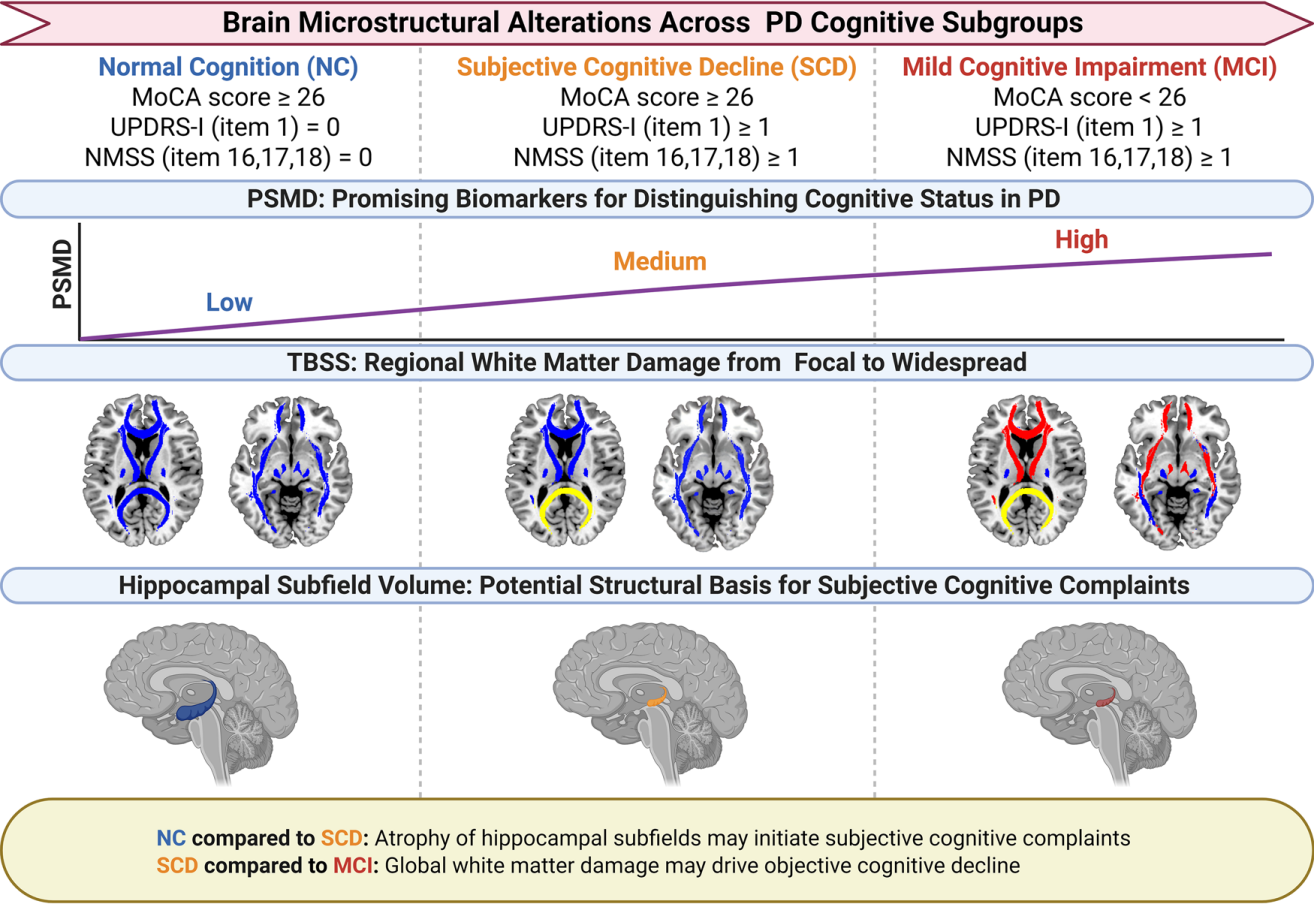
Cross-sectional schematic diagram of brain microstructural changes among different cognitive staging in PD as revealed by neuroimaging. PD Parkinson’s Disease, NC normal cognition, SCD subjective cognitive decline, MCI mild cognitive impairment, NMSS The Non-Motor Symptoms Scale for Parkinson’s disease, PSMD Peak width of Skeletonized Mean Diffusivity, TBSS Tract-Based Spatial Statistics.

This cross-sectional study employed multi-modal neuroimaging to systematically investigate microstructural alterations in PD patients with subjective cognitive complaints for the first time. We observed concurrent white matter microstructural damage (evidenced by increased PSMD and reduced FA in specific tracts) and hippocampal subfield atrophy in patients operationally defined as PD-SCD. These findings provide preliminary neuroimaging correlates of subjective cognitive complaints in PD, offering potential insights into early cognitive changes in this population. However, it should be noted that several key findings in the PD-SCD group were close to the conventional significance threshold of 0.05 (e.g., PSMD difference vs. PD-NC: *p* = 0.035; FA-FMajor vs. HC: *p* = 0.048; HATA volume vs. PD-NC: *p* = 0.037). This may be partly attributable to the heterogeneity of PD-SCD, which can be categorized into rapidly progressive and stable subtypes^1,24^, as well as possible differences in brain microstructure between these two subgroups that need to be confirmed in future longitudinal studies. Consequently, all interpretations in this study should be considered at the group level. A schematic summary of these cross-sectional associations is presented in Fig. 3^25^.

PSMD exhibited a statistically significant cross-sectional gradient across the four groups: PD-MCI > PD-SCD > PD-NC = HC, indicating a pattern of white matter injury that corresponds to cognitive stage. Furthermore, PSMD showed a significant negative correlation with total MoCA scores and its memory and attention subdomains, while a positive correlation was observed with UPDRS-I (item 1) scores. An ordinal logistic regression analysis indicated that PSMD was a strong, significant discriminator in classifying PD cognitive subgroups.

It should be noted that PSMD is a global marker of white matter microstructural injury that may also reflect vascular contributions or other non-PD-specific factors, consistent with previous research wherein PSMD had also been closely linked to AD cognitive impairment^20^. This study further confirms the utility of PSMD in stratifying cognitive stages in PD. Prior studies had also reported that elevated PSMD in PD was associated with reduced dopamine transporter availability in the caudate nucleus (a key region implicated in cognitive processing)^26–28^, which may partly explain the strong correlation between PSMD and cognitive deterioration observed here. Additionally, a previous DTI study reported that widespread elevation of MD value throughout the brain in PD-MCI was significantly correlated with lower cognitive scores^29^. PSMD employed in our study provided a more comprehensive and refined complement to these earlier findings.

Notably, PSMD also differentiated PD-SCD from both PD-NC and HC, suggesting that microstructural white matter alterations are detectable at the stage of subjective cognitive complaints, even in the absence of objective cognitive deficits on standard tests^30^. This finding provides cross-sectional neuroimaging support for the concept that white matter changes may accompany early subjective cognitive concerns in PD.

Thus, PSMD provides a neuroimaging correlate for subjective cognitive complaints in PD, supporting further investigation into whether such complaints may reflect underlying white matter alterations. The cross-sectional gradient in PSMD across groups is consistent with a hypothetical progression model, though longitudinal studies are required to validate this trajectory. In summary, PSMD shows potential as a marker for stratifying cognitive stages in PD in cross-sectional analyses and warrants further investigation in longitudinal and intervention studies.

Then, TBSS analysis was employed to characterize regional white matter alterations linked to cognitive stages in PD, complementing the global white matter injury captured by PSMD. Compared to the PD-NC and HC groups, the PD-MCI group showed significantly reduced FA across multiple white matter fibers, and the FA values of these white matter fibers were significantly positively correlated with cognitive performance, particularly with memory subscores. However, the PD-SCD group exhibited FA reductions limited in the FMajor relative to the HC group, reaching only marginal significance (*p* = 0.048).

Firstly, white matter damage in PD-MCI followed a selective pattern, affecting fiber tracts linked to higher-order cognition. Specifically, widespread FA reductions occurred in SLF, IFOF, UNF, FMajor, and FMinor, which were closely associated with attention, memory integration, language, visual processing, and inter-lobar information transfer^16^, consistent with previous DTI studies on cognitive impairment in PD^17,18,31–35^. ATR, connecting the prefrontal cortex and thalamus, is essential for executive function networks. While ATR FA reduction is seldom reported in PD-MCI, it was associated with executive dysfunction in schizophrenia research^36^. Then, given thalamic atrophy in PD-MCI^37,38^, we proposed that ATR integrity may be compromised.

In the PD-SCD of our cross-sectional sample, white matter alterations remain relatively focal, primarily involving the FMajor, which are critical for visual processing and memory integration^17,31^. This may underlie initial subjective memory complaints. When it comes to the PD-MCI group, white matter damage extended to anterior tracts supporting higher-order executive functions, complex attention, and emotional processing^16^, possibly correlating with transition from memory impairment to multi-domain cognitive impairment. This finding supports Braak’s hypothesis of posterior-to-anterior pathological propagation in PD^39^, though longitudinal data are needed to confirm whether this represents a true temporal progression.

Crucially, compared with the strong discriminative ability of PSMD between PD-SCD and PD-NC, the FA difference in the FMajor was observed only between PD-SCD and HC, reaching merely marginal significance. This suggested that superimposed global white matter damage may contribute more substantially to subjective cognitive complaints, whereas FMajor impairment may be only relatively more pronounced at this stage. In other words, the limited sensitivity of TBSS in distinguishing PD-SCD from PD-NC in our sample may reflect the methodological characteristics of tract-based analyses. TBSS primarily captures focal and tract-specific alterations, which may be insufficient to detect subtle and spatially diffuse microstructural changes at the early stage of subjective cognitive decline. In contrast, PSMD reflects global white matter integrity and may therefore be more sensitive to superimposed and widespread microstructural abnormalities. This methodological difference may partly explain the discrepancy between TBSS and PSMD findings in PD-SCD.

However, ordinal regression including all significant FA values showed that only bilateral IFOF FA independently predicted cognitive staging in PD. Although the full TBSS-FA model was significant and yielded a Nagelkerke pseudo-R² of 0.384, most fibers did not contribute independently. This suggests that while TBSS can detect group-wise differences in multiple tracts, PSMD may offer a more parsimonious and globally sensitive marker for cross-sectional cognitive staging in PD.

Our findings indicate that hippocampal subfield atrophy is already detectable in PD patients with subjective cognitive complaints relative to those without complaints. Several subfields, including CA1-head, GMD-head, and HATA were significantly reduced in PD-SCD compared to PD-NC, but did not differ between PD-SCD and PD-MCI in this cross-sectional sample. This pattern suggests that hippocampal structural changes may occur early in the context of subjective complaints, though we cannot conclude whether atrophy plateaus thereafter, due to sample size limitations, potential floor effects, and the cross-sectional design.

To our knowledge, this is among the first studies to report multi-subfield hippocampal atrophy in PD patients with subjective cognitive complaints. Atrophy in regions such as CA1 and HATA, which are critical for memory encoding, spatial navigation, and emotional regulation, was evident in PD-SCD relative to PD-NC, whereas whole hippocampal volume did not differ^40,41^. This underscores the sensitivity of subfield-level analysis for detecting early structural changes. However, it is important to consider that hippocampal atrophy—particularly in CA1—is also a hallmark of AD pathology^42^. A growing number of studies have suggested that the AD risk gene APOE can also be detected in PD, and that AD-related pathologies such as amyloid accumulation may occur in the early stages of PD^43,44^. Therefore, our findings may reflect mixed AD co-pathology in some PD-SCD patients, a possibility that should be explored in future studies incorporating AD biomarkers.

Although hippocampal subfield atrophy is well-documented in PD-MCI^23,45,46^, its presence in PD-SCD has not been emphasized. Previous SCD studies reported mixed results on hippocampal subfield volumes^47,48^, though some reported no significant changes^49,50^, and most SCD research focuses on AD rather than PD^30^.

The absence of significant volumetric differences between PD-SCD and PD-MCI should be interpreted with caution. While it may suggest that hippocampal atrophy occurs early and does not substantially worsen from the subjective complaint stage to mild cognitive impairment, alternative explanations include measurement precision of automated segmentation and the cross-sectional nature of the data^47,48^. The transition from subjective complaints to objective impairment may involve functional compensation or network dysfunction, such as within the default mode network, which includes the hippocampus^51–54^, rather than further volumetric loss, a hypothesis that could be tested in future longitudinal functional MRI studies. Integrating our multimodal findings, hippocampal subfield atrophy appears to be an early feature in PD patients with cognitive complaints, while white matter alterations—particularly as captured by PSMD—show a clearer cross-sectional gradient from PD-NC to SCD to MCI. This pattern suggests that hippocampal structural changes associated with subjective memory complaints, whereas white matter injury may contribute to objective cognitive impairment. However, this interpretation remains speculative without longitudinal validation.

It must be acknowledged that the cross-sectional nature of our study limits any firm conclusion about a halt in hippocampal atrophy. First, a potential floor effect might be at play, where the volumetric measures of these specific subfields in the PD-SCD group may have already approached a lower limit beyond which further detectable atrophy is limited by the sensitivity of our 3 T MRI sequence or the inherent structural minimal size of these regions. Second, the precision of automated segmentation for delineating hippocampal subfields, particularly in populations with neurodegenerative diseases where anatomical boundaries may be less distinct, might have limited our ability to detect subtle but significant volumetric differences between the PD-SCD and PD-MCI groups. While our findings indicate that significant atrophy could be observed at the SCD stage, future studies with longitudinal designs utilizing ultra-high-field MRI, such as 5 T or 7 T, and even more precise segmentation methods are warranted to confirm the true trajectory of atrophy into the MCI stage.

HATA volume positively correlated with memory scores, and negatively with UPDRS-I (item 1), indicating a distinctive role in PD cognitive impairment. As a transitional region between the hippocampus and amygdala that is implicated in emotional and memory processing, the observed association between HATA volume and these clinical measures is of interest. This pattern is consistent with the clinical observation in PD patients with subjective cognitive complaints, where memory concerns and mood symptoms often co-occur^45,55,56^. However, while it is plausible that structural alterations in the HATA may contribute to explaining the interplay between subjective cognitive and affective symptoms in PD, further longitudinal and multimodal studies are needed to clarify whether HATA atrophy plays a causal role in this comorbidity or reflects a shared underlying pathology.

Multivariate ordinal regression incorporating three hippocampal subfield volumes demonstrated significant efficacy in differentiating among PD cognitive subgroups. Within this model, the volume of the HATA emerged as an independent predictor, suggesting a potentially specific role for this limbic transitional region in cross-sectional cognitive staging. Nevertheless, its independent contribution was modest and should be interpreted with caution, particularly given the borderline *p*-value (*p* = 0.049) and the model’s limited ability to finely distinguish adjacent stages such as PD-SCD from PD-MCI. These findings suggest that hippocampal subfield volumetry, particularly involving HATA, may provide partial role for cognitive status stratification, but its utility for precise staging may be constrained, possibly due to the early occurrence of atrophy and limitations in sample size and measurement.

This study has several limitations: (1) The cross-sectional precludes causal or temporal inferences about the relationship between imaging changes and cognitive decline. Longitudinal studies are essential to track progression from PD-NC to SCD to MCI/dementia; (2) Operational definition of PD-SCD relied primarily on the UPDRS-I (item 1) and NMSS (item 16,17,18), without using validated multidimensional SCD questionnaires, such as Subjective Cognitive Decline Questionnaire, Cognitive Complaints Interview, or Everyday Cognition scale^57^. The absence of these detailed SCD questionnaires may have reduced the sensitivity of TBSS to detect more widespread microstructural alterations by introducing classification noise, potentially explaining the discrepancy with the PSMD findings. Furthermore, we did not distinguish between persistent and intermittent complaints, although we asked patients whether they had frequent cognitive complaints within the past month. This is a relevant consideration, as persistent SCD appears to be more strongly associated with objective cognitive decline and future conversion to MCI or dementia^58^; (3) The sample size, though adequate for group comparisons, is moderate, limiting multivariate power and generalizability. Larger multi-center cohorts are required for validation; (4) Single-center recruitment and exclusion of low-education or comorbid patients may restrict generalizability to broader PD populations; (5) Despite the absence of significant differences in mood scores (e.g., HAM-D, HAM-A) and the implementation of a strict 12-hour medication withdrawal, the potential influence of mood disorders and medication effects on the results could not be entirely ruled out; (6) The study focused on structural markers (DTI/volumetry). Functional MRI or molecular imaging could provide further insights into network dysfunction; (7) Despite quality control, FreeSurfer segmentation may still be affected by partial volume effects and anatomical variability, especially in atrophic regions; and (8) Although we excluded patients with additional neurological conditions, such as severe cerebral small vessel disease, during enrollment, the potential influence of vascular factors or white matter hyperintensities on PSMD cannot be entirely ruled out.

In conclusion, this cross-sectional multimodal neuroimaging study reveals that white matter microstructural alterations (captured by PSMD and TBSS) and hippocampal subfield atrophy are detectable in PD patients with subjective cognitive complaints, even in the absence of objective cognitive impairment. PSMD showed a clear gradient across cognitive stages and strong associations with cognitive performance, supporting its potential utility as a sensitive marker of white matter injury in PD cognition. TBSS suggested a focal-to-widespread pattern of white matter involvement, while hippocampal subfield atrophy appeared early and did not further differentiate SCD from MCI in this sample. These findings provide neuroimaging correlates for subjective cognitive complaints in PD and highlight PSMD as a promising metric for cross-sectional cognitive staging. Future longitudinal studies with validated SCD assessments, AD biomarker profiling, and larger cohorts are needed to clarify the temporal sequence of these changes and their relevance for predicting cognitive progression in PD.

## Methods

### Subjects

68 individuals with PD were prospectively recruited from the Movement Disorders Clinic at Nanjing Medical University’s Affiliated Wuxi People’s Hospital (Wuxi, China) between August 2021 and May 2023. A control group of 27 age- and sex-matched healthy controls (HC) was also included. The study adhered to the ethical principles outlined in the Declaration of Helsinki and received approval from the hospital’s Ethics Committee (Ethical approval number: KY21133). Written informed consent was obtained from all participants prior to enrollment.

PD patient eligibility requirements: (1) Clinical diagnosis of PD based on established criteria^59^; (2) Age at enrollment exceeding 40 years to exclude early-onset PD patients^60^; (3) Right-handedness; and (4) Provision of signed informed consent.

PD patient exclusion criteria for: (1) Prior significant head injury or neurological/psychiatric conditions; (2) Contraindications to MRI or clinical assessments; (3) Structural abnormalities identified on standard MRI; (4) Education level below primary school; and (5) Inadequate imaging quality for analytical processing.

### Clinical features

Following MRI test, comprehensive demographic and clinical data—such as age, gender, education level, disease duration, and LEDD—were collected for all PD participants. Two neurologists with extensive expertise in movement disorders evaluated the PD patients using standardized scales, including: (1) Motor and functional assessments: UPDRS Parts II and III, H&Y Scale, NMSS; (2) Cognitive evaluations: UPDRS Parts Ⅰ (item 1), NMSS, MoCA, FAB; (3) Neuropsychiatric measures: HAM-D and HAM-A. HCs underwent the same set of assessments to ensure comparability.

To precisely characterize domain-specific cognitive decline patterns of subjects, MoCA scores were stratified into four cognitive subdomains for granular assessment: (1) ‘MoCA-visuospatial/executive’ consist of the trail-making, cube copying, and clock copying items, (2) ‘MoCA-memory’ consist of delayed recall and orientation items, (3) ‘MoCA-attention’ consist of attention and language items, (4) ‘MoCA-language’ consist of naming, language and abstraction items, consistent with established approaches in neurodegenerative research^61,62^.

### Grouping of PD patients based on cognitive function

Firstly, PD cognitive grouping was performed according to previous PD-SCD research base on MoCA score and item 1 (intellectual impairment) of UPDRS-I (mentation, behavior, and mood section)^9,10,63,64^.

Subsequently, to further ensure the accuracy of SCD group enrollment, we asked PD patients and their accompanying family members the following three cognitive questions included in the NMSS and documented^24^. Patients were excluded from PD-SCD if they answered ‘no’ to all questions: NMSS item 16: ‘In the past month, has the patient often had difficulty concentrating or staying focused while doing things such as talking or reading?’; NMSS item 17: ‘In the past month, has the patient often forgotten things you just said or things that happened a few days ago?’; NMSS item 18: ‘In the past month, has the patient often forgotten to do certain things, such as taking medication or turning off household appliances?’ We asked both patients and their family members to self-report on the severity and frequency (within the past month) of these issues. Severity was scored as: 0 = asymptomatic, 1 = mild, 2 = moderate, 3 = severe. Frequency was scored as: 1 = less than once per week, 2 = once per week, 3 = several times per week, 4 = daily. Finally, we recorded a composite score as NMSS (item 16,17,18) calculated as severity multiplied by frequency.

PD participants were classified into three groups:

PD-MCI: MoCA score < 26, UPDRS-I (item 1) ≥ 1, and NMSS (item 16,17,18) ≥ 1;

PD-SCD: MoCA score ≥ 26, UPDRS-I (item 1) ≥ 1, and NMSS (item 16,17,18) ≥ 1;

PD-NC: MoCA score ≥ 26, UPDRS-I (item 1) = 0, and NMSS (item 16,17,18) = 0.

### MRI data acquisition

All subjects were scanned using a 3.0 Tesla Siemens Prisma MRI system (Germany) equipped with a 20-channel head coil during the morning. To reduce motion-related distortions and scanner noise, participants were provided with foam cushioning and noise-reducing headphones before image acquisition. PD patients completed their MRI scans during the OFF medication phase after abstaining from dopaminergic medications for 12 h. All participants completed standard clinical MRI protocols along with research-specific sequences (3D T1-weighted imaging and DTI).

The parameters of the 3D-T1 MP-RAGE sequence were as follows: repetition time (TR) = 2300 ms; echo time (TE) = 2.98 ms; inversion time (TI) = 900 ms; flip angle (FA) = 9°; slice thickness = 1 mm; field of view (FOV) = 256 × 256 × 192 mm³; matrix size = 256 × 256; voxel resolution = 1 × 1 × 1 mm³; and a total acquisition time (TA) of 5 min 30 s.

The parameters of the DTI sequence were: TR = 4100 ms; TE = 72 ms; FOV = 210 × 210 mm²; diffusion directions = 64; b-value = 1000 s/mm^2^; slice thickness = 2 mm; voxel size = 2 × 2 × 2 mm³; and a total TA of 5 min 18 s.

### MRI data post-processing

DTI data was preprocessed by FMRIB Software Library (FSL, version 6.0.1, http://www.fmrib.ox.ac.uk/fsl), with the following pipeline: (1) Dicom-to-NIfTI conversion using dcm2niix; (2) eddy current & motion correction; (3) brain extraction; and (4) FA and MD maps were generated by tensor fitting^20^.

TBSS processing pipeline was implemented as follows: (1) all individual FA maps were registered to FMRIB152 template to attain normalized individual FA maps; (2) generating mean FA map by averaging all normalized individual FA maps; (3) creating mean FA skeleton map and mean FA skeleton mask (Thresholded at FA > 0.2); (4) projecting normalized individual FA maps onto mean FA skeleton mask to generate individual skeletonized FA maps; (5) tract-specific FA values were extracted from each individual skeletonized FA map using the JHU White-Matter Tractography Atlas, including bilateral ATR, bilateral CST, bilateral cingulum-cingulate gyrus (CGC), bilateral cingulum-hippocampus (CGH), FMajor, FMinor, bilateral IFOF, bilateral ILF, bilateral SLF, and bilateral UNF^65,66^.

PSMD analysis was conducted according to the following pipeline (version 1.8.2; https://github.com/miac-research/psmd/releases): (1) applying the same spatial transformation parameters derived from the TBSS-based FA skeletonization on MD maps to obtain mean MD skeleton map and mean MD skeleton mask (Thresholded at FA > 0.3 and masked with white matter masks derived from TBSS skeletonization to further minimize cerebrospinal fluid partial volume effects); (2) projecting normalized individual MD maps onto the mean MD skeleton mask to generate individual skeletonized MD maps; (3) PSMD metric was then defined as the histogram width between the 95th and 5th percentiles of MD values across all skeleton voxels^20,67^.

High-resolution T1-weighted structural MRI scans were processed using the automated recon-all pipeline within FreeSurfer software (version 7.4.1, http://surfer.nmr.mgh.harvard.edu). This comprehensive pipeline performs several key preprocessing steps, including: intensity normalization to correct for scanner inhomogeneities, skull stripping to remove non-brain tissue, affine Talairach transformation for spatial normalization, and automated volumetric segmentation of subcortical gray matter structures and cortical parcellation.

Following the initial recon-all processing, the preprocessed T1-weighted images were specifically subjected to segmentation of the hippocampus and its constituent subfields using dedicated hippocampal subfield module in FreeSurfer (version 7.4.1). This module has demonstrated excellent test-retest reliability and robustness to variations in MRI acquisition parameters. The segmentation procedure delineates the hippocampus into the following 19 distinct subregions bilaterally: CA1-head/body, CA3-head/body, CA4-head/body, GMD-head/body, molecular layer (ML)-head/body, subiculum (SIL)-head/body, presubiculum (PSIL)-head/body, parasubiculum (PARA), HATA, fimbria, tail, and fissure. Additionally, we extracted the total intracranial volume (TIV) to account for overall brain size differences.

All automated segmentations, including the hippocampal subfields and whole-brain structures, underwent rigorous visual inspection in both sagittal and coronal views by two senior neuroradiologists with extensive expertise (blinded to group status/experimental conditions) to identify and, if necessary, manually correct any gross segmentation errors according to established protocols. Ultimately, no manual correction was required for any of the segmentation. Volumetric measures for each hippocampal subfield were computed based on the probabilistic segmentation outputs (“soft segmentation”). The final volumes of hippocampal subfields were expressed as a ratio to TIV in ten-thousandths (i.e., [volume/TIV]×10⁴) to control for individual differences in head size.

### Statistical analysis

Statistical analyses were carried out with IBM SPSS Statistics Version 26.0. A *p*-value of < 0.05 was considered statistically significant, and the Bonferroni method was used to adjust for all multiple comparisons where appropriate. Continuous demographic and clinical variables are presented as mean ± standard deviation.

Data normality was evaluated using the Shapiro–Wilk test. Continuous clinical variables were compared across groups via ANOVA analysis, with post hoc two-sample t-tests for significant findings, while categorical variables were analyzed with the Chi-square test. Subsequently, account for the potential confounding effects of age, sex, education level, medication, and mood, two sets of ANCOVA analyses were applied to research the difference of neuroimaging markers among groups. Among PD patient subgroups, ANCOVA was conducted with age, sex, years of education, LEDD, HAMD, and HAMA scores included as covariates; since LEDD is not applicable to HC group, ANCOVA was performed using only age, sex, years of education, HAMD, and HAMA scores as covariates across all four groups. This approach allows for a comparison between PD and HC while adjusting for common demographic, drug- and mood-related factors. Regarding to neuroimaging markers, post hoc two-sample t-tests were conducted for only those variables that showed significant differences in both two sets of ANCOVA analyses. For ANOVA or ANCOVA, η² was reported as the effect size, and for key post hoc tests, Cohen’s d with its 95% confidence interval (CI) was provided. Partial correlation analyses were conducted to examine the relationships between clinical features and neuroimaging markers within PD patients including age, gender, years of education, LEDD, HAMD, and HAMA as covariates. Finally, adjusting for the same covariates as in partial correlation analysis, ordinal logistic regression analyses were conducted for PD subjects to evaluate the utility of each neuroimaging biomarker (PSMD, TBSS, and hippocampal subfield volume) in differentiating cognitive stages in PD. All variables showing significant differences across all four groups and among PD subgroups simultaneously were included in the model. All analyzed variables had undergone Z-value normalization to eliminate dimensional influence and facilitate comparison of effect sizes, and the model had passed all necessary hypothesis tests. For multivariable models that reached statistical significance, variance inflation factor (VIF) checks were performed for collinearity diagnostics.

## Supplementary information

Supplementary Information

## Data Availability

The datasets generated and/or analyzed during the current study are not publicly available due to ethical guidelines on patient privacy rights, but are available from the corresponding author on reasonable request.
