# Supplementary material for: Neuroimaging evidence of microstructural alteration in Parkinson’s disease with subjective cognitive decline
Source: NPJ Parkinsons Dis. 2026 Mar 13;12:105. doi: 10.1038/s41531-026-01313-y (PMC13128858; doi:10.1038/s41531-026-01313-y)
Supplement: Supplementary file 1 — Supplementary Information [file 41531_2026_1313_MOESM1_ESM.pdf]

Supplementary Figure S1

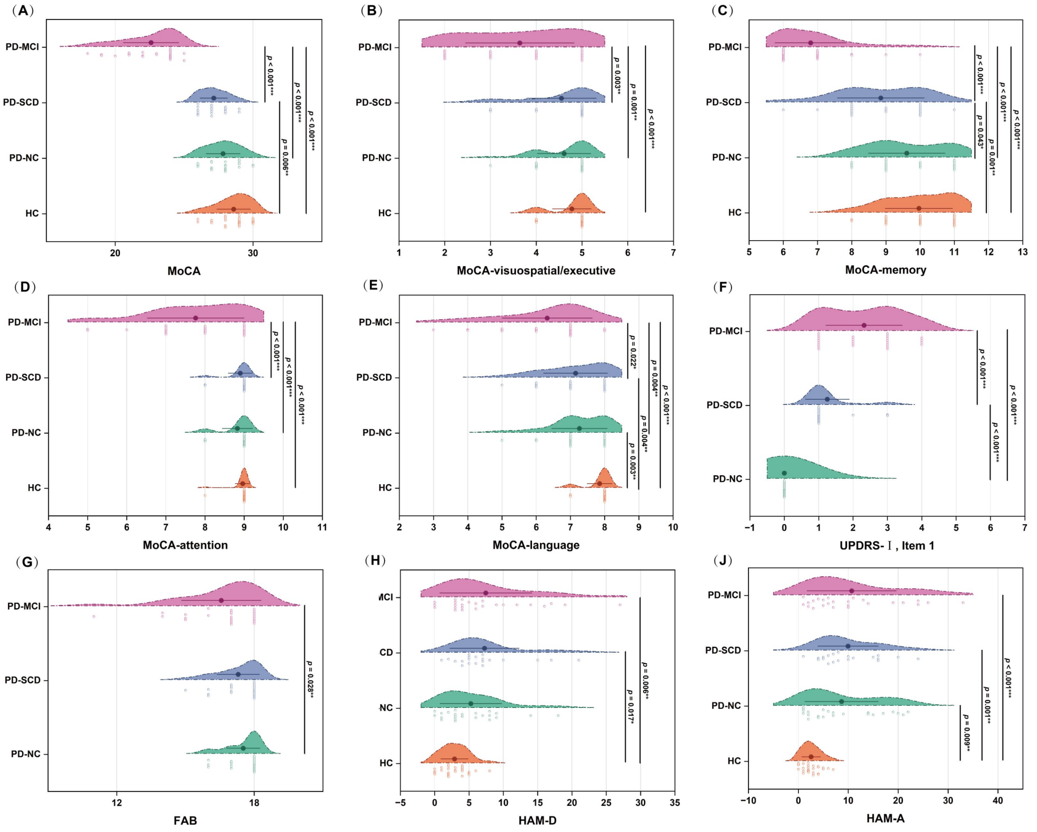

Supplementary Figure S1. Comparison of clinical features among PD-MCI (N = 25), PD-SCD (N = 20), PD-NC (N = 23) and HC (N = 27) with ANOVA analysis. (A) MoCA; **(B)** MoCA-visuospatial/executive; **(C)** MoCA-memory; **(D)** MoCA-attention; **(E)** MoCA-language; **(F)** UPDRS-I, Item 1; **(G)** FAB; **(H)** HAM-D**; (I)** HAM-A. Error bars represent the standard error of the mean. Asterisks indicate statistically significant group differences (*: $p < 0.05$, **: $p < 0.01$,***: $p < 0.01$). Abbreviations: PD, Parkinson's disease; MCI, mild cognitive impairment; SCD, subjective cognitive decline; NC, non cognitive impairment; HC, healthy control; MoCA, Montreal Cognitive Assessment; UPDRS, Unified Parkinson's Disease Rating Scale; FAB, Frontal Assessment Battery; HAM-D, 17-item Hamilton Depression Rating Scale; HAM-A, Hamilton Anxiety Rating Scale.

Supplementary Table S1 Partial correlations between clinical features and neuroimaging biomarkers in patients with Parkinson's disease adjusted for age, gender, education, HAM-D, HAM-A, and LEDD

	MoCA	MoCA-visuospatial/executive	MoCA-memory	MoCA-attention	MoCA-language	FAB	UPDRS-I, Item 1	UPDRS-II	UPDRS-III
**PSMD**	-0.401<br>[-0.601, -0.158]<br>(0.001***)	-0.125<br>[-0.380, 0.144]<br>(0.333)	-0.416<br>[-0.614, -0.176]<br>(0.001***)	-0.287<br>[-0.531, -0.011]<br>(0.023*)	-0.185<br>[-0.459, 0.112]<br>(0.15)	0.056<br>[-0.209, 0.311]<br>(0.665)	0.324<br>[0.050, 0.552]<br>(0.01**)	0.042<br>[-0.224, 0.302]<br>(0.743)	0.094<br>[-0.179, 0.352]<br>(0.468)
**TBSS-FA**									
**ATR$_L$**	0.310<br>[0.041, 0.537]<br>(0.014*)	0.081<br>[-0.188, 0.341]<br>(0.533)	0.317<br>[0.051, 0.542]<br>(0.012*)	0.162<br>[-0.109, 0.414]<br>(0.209)	0.193<br>[-0.083, 0.442]<br>(0.133)	0.002<br>[-0.263, 0.266]<br>(0.985)	-0.247<br>[-0.503, 0.045]<br>(0.053)	0.035<br>[-0.230, 0.297]<br>(0.787)	0.078<br>[-0.193, 0.339]<br>(0.545)
**ATR$_R$**	0.346<br>[0.083, 0.566]<br>(0.006**)	0.144<br>[-0.123, 0.395]<br>(0.266)	0.321<br>[0.055, 0.545]<br>(0.011*)	0.276<br>[0.006, 0.514]<br>(0.03*)	0.170<br>[-0.096, 0.416]<br>(0.187)	0.008<br>[-0.258, 0.273]<br>(0.948)	-0.293<br>[-0.541, -0.012]<br>(0.021*)	-0.036<br>[-0.303, 0.233]<br>(0.783)	-0.002<br>[-0.267, 0.264]<br>(0.985)
**CST$_L$**	0.171<br>[-0.097, 0.420]<br>(0.184)	0.017<br>[-0.248, 0.279]<br>(0.898)	0.174<br>[-0.093, 0.421]<br>(0.175)	0.029<br>[-0.239, 0.292]<br>(0.821)	0.242<br>[-0.026, 0.485]<br>(0.058)	-0.052<br>[-0.321, 0.222]<br>(0.686)	-0.024<br>[-0.294, 0.249]<br>(0.852)	-0.102<br>[-0.373, 0.180]<br>(0.432)	-0.140<br>[-0.405, 0.143]<br>(0.278)
**CST$_R$**	0.353<br>[0.091, 0.572]<br>(0.005**)	0.090<br>[-0.175, 0.348]<br>(0.487)	0.268<br>[-0.002, 0.506]<br>(0.036*)	0.231<br>[-0.041, 0.475]<br>(0.071)	0.368<br>[0.108, 0.585]<br>(0.003**)	0.085<br>[-0.185, 0.346]<br>(0.509)	-0.154<br>[-0.423, 0.131]<br>(0.232)	-0.110<br>[-0.378, 0.172]<br>(0.395)	-0.112<br>[-0.382, 0.169]<br>(0.385)
**CGC$_L$**	0.143<br>[-0.128, 0.397]<br>(0.269)	0.009<br>[-0.257, 0.274]<br>(0.948)	0.099<br>[-0.168, 0.356]<br>(0.444)	0.203<br>[-0.064, 0.448]<br>(0.114)	0.176<br>[-0.093, 0.424]<br>(0.172)	0.023<br>[-0.245, 0.287]<br>(0.862)	-0.063<br>[-0.335, 0.218]<br>(0.626)	0.214<br>[-0.055, 0.461]<br>(0.094)	0.182<br>[-0.082, 0.430]<br>(0.156)
**CGC$_R$**	0.051<br>[-0.219, 0.315]<br>(0.694)	-0.060<br>[-0.327, 0.214]<br>(0.643)	0.045<br>[-0.223, 0.309]<br>(0.73)	0.077<br>[-0.196, 0.342]<br>(0.554)	0.143<br>[-0.126, 0.396]<br>(0.266)	-0.093<br>[-0.361, 0.184]<br>(0.474)	0.058<br>[-0.214, 0.325]<br>(0.652)	0.196<br>[-0.074, 0.446]<br>(0.127)	0.159<br>[-0.117, 0.417]<br>(0.218)
**CGH$_L$**	0.038<br>[-0.233, 0.304]<br>(0.771)	0.039<br>[-0.229, 0.303]<br>(0.766)	0.028<br>[-0.244, 0.298]<br>(0.827)	-0.012<br>[-0.283, 0.261]<br>(0.928)	0.087<br>[-0.184, 0.350]<br>(0.504)	0.070<br>[-0.203, 0.336]<br>(0.587)	-0.096<br>[-0.368, 0.187]<br>(0.457)	0.153<br>[-0.124, 0.410]<br>(0.236)	0.241<br>[-0.024, 0.482]<br>(0.059)
**CGH$_R$**	0.180<br>[-0.085, 0.426]<br>(0.161)	0.002<br>[-0.265, 0.269]<br>(0.991)	0.112<br>[-0.156, 0.371]<br>(0.388)	0.110<br>[-0.161, 0.371]<br>(0.395)	0.229<br>[-0.043, 0.477]<br>(0.074)	-0.015<br>[-0.283, 0.254]<br>(0.909)	-0.100<br>[-0.368, 0.181]<br>(0.441)	-0.014<br>[-0.286, 0.259]<br>(0.913)	0.036<br>[-0.233, 0.300]<br>(0.783)
**FMajor**	0.223<br>[-0.045, 0.467]<br>(0.081)	-0.021<br>[-0.287, 0.247]<br>(0.874)	0.331<br>[0.071, 0.553]<br>(0.009**)	0.115<br>[-0.154, 0.375]<br>(0.373)	0.032<br>[-0.236, 0.296]<br>(0.802)	-0.140<br>[-0.405, 0.141]<br>(0.278)	-0.262<br>[-0.511, 0.023]<br>(0.039*)	0.103<br>[-0.164, 0.361]<br>(0.426)	0.100<br>[-0.168, 0.359]<br>(0.442)
**FMinor**	0.329<br>[0.067, 0.552]<br>(0.009**)	0.095<br>[-0.173, 0.354]<br>(0.461)	0.320<br>[0.054, 0.547]<br>(0.011*)	0.222<br>[-0.046, 0.467]<br>(0.082)	0.252<br>[0.000, 0.481]<br>(0.048*)	0.040<br>[-0.229, 0.304]<br>(0.759)	-0.196<br>[-0.449, 0.080]<br>(0.127)	0.034<br>[-0.235, 0.299]<br>(0.792)	-0.041<br>[-0.308, 0.229]<br>(0.749)
**IFOF$_L$**	0.353	0.088	0.372	0.194	0.184	0.007	-0.270	0.049	-0.008

	[0.091, 0.572]<br>(0.005**)	[-0.179, 0.346]<br>(0.497)	[0.113, 0.587]<br>(0.003**)	[-0.080, 0.445]<br>(0.132)	[-0.091, 0.438]<br>(0.152)	[-0.260, 0.273]<br>(0.96)	[-0.519, 0.013]<br>(0.034*)	[-0.223, 0.314]<br>(0.704)	[-0.277, 0.261]<br>(0.952)
**IFOF$_R$**	0.276<br>[0.006, 0.514]<br>(0.03*)	0.049<br>[-0.221, 0.315]<br>(0.705)	0.308<br>[0.040, 0.539]<br>(0.015*)	0.116<br>[-0.154, 0.375]<br>(0.371)	0.175<br>[-0.100, 0.431]<br>(0.174)	-0.060<br>[-0.328, 0.216]<br>(0.643)	-0.217<br>[-0.470, 0.060]<br>(0.09)	0.034<br>[-0.237, 0.299]<br>(0.792)	0.038<br>[-0.234, 0.303]<br>(0.768)
**ILF$_L$**	0.178<br>[-0.087, 0.425]<br>(0.166)	-0.022<br>[-0.289, 0.248]<br>(0.865)	0.208<br>[-0.060, 0.454]<br>(0.105)	0.128<br>[-0.139, 0.386]<br>(0.32)	0.172<br>[-0.098, 0.424]<br>(0.181)	-0.102<br>[-0.373, 0.180]<br>(0.431)	-0.110<br>[-0.379, 0.170]<br>(0.393)	0.098<br>[-0.174, 0.361]<br>(0.447)	0.074<br>[-0.197, 0.338]<br>(0.568)
**ILF$_R$**	0.239<br>[-0.029, 0.484]<br>(0.061)	-0.011<br>[-0.279, 0.258]<br>(0.934)	0.306<br>[0.037, 0.539]<br>(0.016*)	0.134<br>[-0.133, 0.391]<br>(0.301)	0.091<br>[-0.180, 0.353]<br>(0.483)	-0.087<br>[-0.353, 0.189]<br>(0.502)	-0.131<br>[-0.399, 0.155]<br>(0.308)	-0.012<br>[-0.282, 0.259]<br>(0.923)	-0.055<br>[-0.324, 0.221]<br>(0.668)
**SLF$_L$**	0.303<br>[0.033, 0.537]<br>(0.017*)	-0.037<br>[-0.304, 0.234]<br>(0.773)	0.373<br>[0.115, 0.589]<br>(0.003**)	0.190<br>[-0.083, 0.442]<br>(0.139)	0.262<br>[0.010, 0.492]<br>(0.04*)	-0.016<br>[-0.285, 0.255]<br>(0.901)	-0.183<br>[-0.440, 0.094]<br>(0.154)	-0.074<br>[-0.344, 0.205]<br>(0.566)	-0.096<br>[-0.364, 0.185]<br>(0.458)
**SLF$_R$**	0.287<br>[0.015, 0.525]<br>(0.024*)	0.042<br>[-0.228, 0.307]<br>(0.747)	0.326<br>[0.064, 0.551]<br>(0.01*)	0.210<br>[-0.056, 0.456]<br>(0.101)	0.187<br>[-0.086, 0.440]<br>(0.146)	0.033<br>[-0.236, 0.298]<br>(0.8)	-0.177<br>[-0.432, 0.100]<br>(0.169)	0.017<br>[-0.255, 0.286]<br>(0.898)	-0.002<br>[-0.270, 0.266]<br>(0.99)
**UNF$_L$**	0.307<br>[0.037, 0.541]<br>(0.015*)	0.078<br>[-0.192, 0.341]<br>(0.548)	0.332<br>[0.073, 0.553]<br>(0.008**)	0.037<br>[-0.236, 0.305]<br>(0.776)	0.246<br>[-0.015, 0.482]<br>(0.053)	0.213<br>[-0.055, 0.461]<br>(0.097)	-0.320<br>[-0.559, -0.047]<br>(0.011*)	-0.026<br>[-0.297, 0.248]<br>(0.841)	0.011<br>[-0.259, 0.279]<br>(0.933)
**UNF$_R$**	0.359<br>[0.099, 0.579]<br>(0.004**)	-0.005<br>[-0.274, 0.265]<br>(0.967)	0.404<br>[0.156, 0.611]<br>(0.001***)	0.102<br>[-0.168, 0.362]<br>(0.428)	0.287<br>[0.015, 0.526]<br>(0.023*)	0.217<br>[-0.049, 0.462]<br>(0.091)	-0.267<br>[-0.515, 0.017]<br>(0.036*)	-0.125<br>[-0.389, 0.155]<br>(0.335)	-0.061<br>[-0.329, 0.215]<br>(0.639)
**Hippocampal Volume**									
**CA1-head**	0.199<br>[-0.069, 0.448]<br>(0.121)	0.064<br>[-0.202, 0.325]<br>(0.62)	0.147<br>[-0.122, 0.401]<br>(0.255)	0.056<br>[-0.212, 0.319]<br>(0.665)	0.143<br>[-0.126, 0.396]<br>(0.266)	0.040<br>[-0.229, 0.304]<br>(0.755)	-0.224<br>[-0.473, 0.051]<br>(0.08)	-0.053<br>[-0.322, 0.222]<br>(0.683)	-0.028<br>[-0.299, 0.245]<br>(0.826)
**CA1-body**	-0.166<br>[-0.424, 0.110]<br>(0.196)	-0.230<br>[-0.484, 0.046]<br>(0.069)	-0.014<br>[-0.284, 0.258]<br>(0.913)	-0.217<br>[-0.470, 0.059]<br>(0.087)	-0.003<br>[-0.272, 0.267]<br>(0.98)	-0.115<br>[-0.381, 0.164]<br>(0.374)	-0.156<br>[-0.425, 0.130]<br>(0.226)	-0.193<br>[-0.449, 0.087]<br>(0.134)	-0.112<br>[-0.381, 0.168]<br>(0.385)
**CA3-head**	-0.036<br>[-0.303, 0.234]<br>(0.78)	-0.103<br>[-0.370, 0.174]<br>(0.424)	0.186<br>[-0.087, 0.439]<br>(0.147)	-0.265<br>[-0.513, 0.019]<br>(0.037*)	-0.164<br>[-0.422, 0.114]<br>(0.203)	0.050<br>[-0.219, 0.315]<br>(0.699)	-0.324<br>[-0.557, -0.054]<br>(0.01*)	-0.071<br>[-0.341, 0.206]<br>(0.585)	0.003<br>[-0.267, 0.273]<br>(0.983)
**CA3-body**	-0.028<br>[-0.299, 0.245]<br>(0.829)	-0.188<br>[-0.443, 0.089]<br>(0.144)	0.106<br>[-0.163, 0.367]<br>(0.413)	-0.171<br>[-0.428, 0.106]<br>(0.185)	0.003<br>[-0.268, 0.273]<br>(0.98)	-0.029<br>[-0.299, 0.245]<br>(0.82)	-0.152<br>[-0.424, 0.135]<br>(0.24)	-0.116<br>[-0.383, 0.163]<br>(0.371)	-0.051<br>[-0.321, 0.225]<br>(0.695)
**CA4-head**	0.022<br>[-0.249, 0.291]<br>(0.867)	-0.063<br>[-0.332, 0.213]<br>(0.629)	0.153<br>[-0.118, 0.407]<br>(0.234)	-0.208<br>[-0.463, 0.068]<br>(0.104)	-0.051<br>[-0.320, 0.224]<br>(0.695)	0.020<br>[-0.249, 0.287]<br>(0.875)	-0.296<br>[-0.540, -0.022]<br>(0.019*)	-0.018<br>[-0.288, 0.254]<br>(0.889)	0.043<br>[-0.227, 0.308]<br>(0.74)

**CA4-body**	-0.048<br>[-0.318, 0.227]<br>(0.708)	-0.111<br>[-0.378, 0.167]<br>(0.392)	0.067<br>[-0.202, 0.331]<br>(0.605)	-0.204<br>[-0.459, 0.072]<br>(0.112)	-0.013<br>[-0.284, 0.259]<br>(0.919)	-0.033<br>[-0.303, 0.240]<br>(0.799)	-0.190<br>[-0.448, 0.089]<br>(0.138)	0.007<br>[-0.264, 0.277]<br>(0.958)	0.103<br>[-0.172, 0.365]<br>(0.424)
**GMD-head**	0.046<br>[-0.225, 0.312]<br>(0.72)	-0.054<br>[-0.322, 0.221]<br>(0.679)	0.175<br>[-0.096, 0.426]<br>(0.173)	-0.153<br>[-0.414, 0.125]<br>(0.234)	-0.059<br>[-0.329, 0.219]<br>(0.648)	0.020<br>[-0.249, 0.288]<br>(0.876)	-0.333<br>[-0.562, -0.072]<br>(0.008**)	-0.057<br>[-0.327, 0.219]<br>(0.659)	0.001<br>[-0.269, 0.271]<br>(0.994)
**GMD-body**	-0.013<br>[-0.285, 0.260]<br>(0.92)	-0.091<br>[-0.360, 0.186]<br>(0.482)	0.118<br>[-0.148, 0.376]<br>(0.359)	-0.196<br>[-0.452, 0.082]<br>(0.127)	-0.018<br>[-0.289, 0.255]<br>(0.89)	-0.077<br>[-0.348, 0.201]<br>(0.551)	-0.238<br>[-0.489, 0.034]<br>(0.063)	-0.029<br>[-0.301, 0.244]<br>(0.82)	0.047<br>[-0.225, 0.313]<br>(0.714)
**ML-head**	0.149<br>[-0.119, 0.403]<br>(0.248)	0.018<br>[-0.252, 0.285]<br>(0.888)	0.139<br>[-0.127, 0.393]<br>(0.282)	0.018<br>[-0.252, 0.286]<br>(0.89)	0.073<br>[-0.197, 0.336]<br>(0.575)	0.002<br>[-0.266, 0.270]<br>(0.986)	-0.194<br>[-0.452, 0.085]<br>(0.13)	0.007<br>[-0.264, 0.277]<br>(0.959)	0.053<br>[-0.219, 0.319]<br>(0.683)
**ML-body**	-0.043<br>[-0.314, 0.232]<br>(0.738)	-0.145<br>[-0.406, 0.130]<br>(0.26)	0.071<br>[-0.200, 0.336]<br>(0.585)	-0.122<br>[-0.385, 0.154]<br>(0.344)	-0.041<br>[-0.312, 0.235]<br>(0.75)	-0.160<br>[-0.428, 0.124]<br>(0.214)	-0.172<br>[-0.435, 0.109]<br>(0.181)	-0.003<br>[-0.274, 0.268]<br>(0.979)	0.080<br>[-0.191, 0.344]<br>(0.538)
**SIL-head**	0.184<br>[-0.090, 0.439]<br>(0.153)	0.084<br>[-0.182, 0.344]<br>(0.518)	0.003<br>[-0.266, 0.272]<br>(0.983)	0.183<br>[-0.090, 0.438]<br>(0.155)	0.179<br>[-0.092, 0.431]<br>(0.163)	-0.085<br>[-0.353, 0.192]<br>(0.512)	0.056<br>[-0.216, 0.323]<br>(0.665)	0.027<br>[-0.246, 0.297]<br>(0.835)	0.058<br>[-0.213, 0.325]<br>(0.654)
**SIL-body**	-0.005<br>[-0.276, 0.266]<br>(0.97)	-0.006<br>[-0.275, 0.264]<br>(0.964)	0.025<br>[-0.247, 0.295]<br>(0.847)	-0.013<br>[-0.284, 0.259]<br>(0.92)	-0.059<br>[-0.330, 0.219]<br>(0.651)	-0.083<br>[-0.353, 0.195]<br>(0.523)	-0.149<br>[-0.415, 0.133]<br>(0.247)	0.114<br>[-0.161, 0.379]<br>(0.378)	0.207<br>[-0.062, 0.455]<br>(0.107)
**PSIL-head**	0.159<br>[-0.108, 0.413]<br>(0.217)	0.087<br>[-0.180, 0.348]<br>(0.502)	0.115<br>[-0.154, 0.375]<br>(0.374)	0.135<br>[-0.133, 0.391]<br>(0.296)	0.042<br>[-0.228, 0.307]<br>(0.746)	0.025<br>[-0.245, 0.293]<br>(0.849)	-0.029<br>[-0.301, 0.245]<br>(0.824)	0.106<br>[-0.163, 0.367]<br>(0.412)	0.152<br>[-0.118, 0.406]<br>(0.238)
**PSIL-body**	0.125<br>[-0.141, 0.382]<br>(0.334)	0.090<br>[-0.178, 0.351]<br>(0.489)	0.102<br>[-0.166, 0.363]<br>(0.431)	0.171<br>[-0.106, 0.429]<br>(0.185)	-0.009<br>[-0.279, 0.262]<br>(0.942)	-0.053<br>[-0.323, 0.223]<br>(0.685)	-0.035<br>[-0.307, 0.241]<br>(0.79)	0.251<br>[0.000, 0.483]<br>(0.049*)	0.268<br>[0.010, 0.501]<br>(0.035*)
**PARA**	0.014<br>[-0.257, 0.283]<br>(0.913)	0.065<br>[-0.205, 0.328]<br>(0.614)	0.028<br>[-0.243, 0.297]<br>(0.828)	0.011<br>[-0.259, 0.280]<br>(0.929)	-0.025<br>[-0.295, 0.248]<br>(0.845)	0.014<br>[-0.256, 0.282]<br>(0.915)	0.142<br>[-0.130, 0.399]<br>(0.272)	0.102<br>[-0.168, 0.365]<br>(0.43)	0.183<br>[-0.091, 0.438]<br>(0.155)
**HATA**	0.227<br>[-0.042, 0.472]<br>(0.076)	0.036<br>[-0.233, 0.302]<br>(0.782)	0.333<br>[0.072, 0.556]<br>(0.008**)	0.065<br>[-0.205, 0.329]<br>(0.614)	-0.037<br>[-0.307, 0.236]<br>(0.776)	0.086<br>[-0.183, 0.348]<br>(0.506)	-0.268<br>[-0.515, 0.015]<br>(0.035*)	-0.188<br>[-0.444, 0.092]<br>(0.144)	-0.126<br>[-0.390, 0.152]<br>(0.328)
**Fimbria**	0.058<br>[-0.212, 0.324]<br>(0.657)	0.125<br>[-0.142, 0.382]<br>(0.335)	0.008<br>[-0.263, 0.279]<br>(0.949)	0.116<br>[-0.157, 0.378]<br>(0.37)	-0.133<br>[-0.395, 0.145]<br>(0.303)	-0.157<br>[-0.425, 0.128]<br>(0.223)	-0.022<br>[-0.295, 0.252]<br>(0.864)	0.166<br>[-0.101, 0.417]<br>(0.196)	0.105<br>[-0.168, 0.368]<br>(0.417)
**Tail**	-0.114<br>[-0.381, 0.165]<br>(0.377)	-0.161<br>[-0.424, 0.119]<br>(0.21)	-0.137<br>[-0.402, 0.141]<br>(0.29)	-0.056<br>[-0.324, 0.218]<br>(0.663)	0.007<br>[-0.265, 0.278]<br>(0.955)	-0.129<br>[-0.397, 0.153]<br>(0.316)	0.020<br>[-0.252, 0.289]<br>(0.876)	-0.019<br>[-0.290, 0.254]<br>(0.883)	0.004<br>[-0.267, 0.274]<br>(0.977)

**Fissure**	-0.011<br>[-0.281, 0.260]<br>(0.931)	-0.214<br>[-0.474, 0.068]<br>(0.095)	0.005<br>[-0.265, 0.275]<br>(0.971)	-0.125<br>[-0.387, 0.151]<br>(0.334)	0.242<br>[-0.026, 0.485]<br>(0.058)	0.072<br>[-0.199, 0.338]<br>(0.578)	-0.050<br>[-0.321, 0.225]<br>(0.7)	-0.122<br>[-0.387, 0.153]<br>(0.344)	0.010<br>[-0.260, 0.279]<br>(0.942)
**Whole hippocampus**	0.046<br>[-0.225, 0.312]<br>(0.725)	-0.055<br>[-0.323, 0.221]<br>(0.671)	0.081<br>[-0.184, 0.340]<br>(0.529)	-0.049<br>[-0.318, 0.226]<br>(0.705)	0.011<br>[-0.260, 0.280]<br>(0.93)	-0.066<br>[-0.334, 0.208]<br>(0.611)	-0.171<br>[-0.434, 0.110]<br>(0.183)	0.004<br>[-0.266, 0.273]<br>(0.977)	0.067<br>[-0.203, 0.332]<br>(0.606)

Results are presented as correlation coefficient [95% CI] (*p*-value).

Abbreviations: HAM-D: Hamilton Depression Rating Scale; HAM-A: Hamilton Anxiety Rating Scale; LEDD, levodopa equivalent daily dose; MoCA, Montreal Cognitive Assessment; FAB, Frontal Assessment Battery; UPDRS, Unified Parkinson's Disease Rating Scale; HAM-D, 17-item Hamilton Depression Rating Scale; HAM-A, Hamilton Anxiety Rating Scale; PSMD, peak width of skeletonized mean diffusivity; ATR, anterior thalamic radiation; CST, corticospinal tract; CGC, cingulum-cingulate gyrus; CGH, cingulum-hippocampus; FMajor, forceps major; FMinor, forceps minor; IFOF, inferior fronto-occipital fasciculus; ILF, inferior longitudinal fasciculus; SLF, superior longitudinal fasciculus; UNF, uncinate fasciculus; $_{L}$, left hemisphere; $_{R}$, right hemisphere; CA, cornu ammonis; GMD, granule cell and molecular layer of the dentate gyrus; ML, molecular layer; SIL, subiculum; PSIL, presubiculum; PARA, parasubiculum; HATA, hippocampal-amygdaloid transition area.

Significance levels: $^{*}p < 0.05$, $^{**}p < 0.01$, $^{***}p < 0.001$.

STROBE statement: Reporting guidelines checklist for cohort, case-control and cross-sectional studies

SECTION	ITEM NUMBER	CHECKLIST ITEM	REPORTED ON PAGE NUMBER:
**TITLE AND ABSTRACT**			
	1a	Indicate the study's design with a commonly used term in the title or the abstract	1-2
	1b	Provide in the abstract an informative and balanced summary of what was done and what was found	2
**INTRODUCTION**			
Background and objectives	2	Explain the scientific background and rationale for the investigation being reported	3-4
	3	State specific objectives, including any pre-specified hypotheses	4
**METHODS**			
Study design	4	Present key elements of study design early in the paper	16
Setting	5	Describe the setting, locations, and relevant dates, including periods of recruitment, exposure, follow-up, and data collection	16
Participants	6a	Cohort study—Give the eligibility criteria, and the sources and methods of selection of participants. Describe methods of follow-up<br>Case-control study—Give the eligibility criteria, and the sources and methods of case ascertainment and control selection. Give the rationale for the choice of cases and controls<br>Cross-sectional study—Give the eligibility criteria, and the sources and methods of selection of participants	16-17
	6b	Cohort study—For matched studies, give matching criteria and number of exposed and unexposed<br>Case-control study—For matched studies, give matching criteria and the number of controls per case<br>Variables	16-17
Variables	7	Clearly define all outcomes, exposures, predictors, potential confounders, and effect modifiers. Give diagnostic criteria, if applicable	16-17
Data sources/measurements	8*	For each variable of interest, give sources of data and details of methods of assessment	16-17

SECTION	ITEM NUMBER	CHECKLIST ITEM	REPORTED ON PAGE NUMBER:
		(measurement). Describe comparability of assessment methods if there is more than one group.	
Bias	9	Describe any efforts to address potential sources of bias.	18
Study size	10	Explain how the study size was arrived at	16
Quantitative variables	11	Explain how quantitative variables were handled in the analyses. If applicable, describe which groupings were chosen and why.	17,21
Statistical methods	12a	Describe all statistical methods, including those used to control for confounding	21-22
	12b	Describe any methods used to examine subgroups and interactions	21
	12c	Explain how missing data were addressed	16
	12d	Cohort study—If applicable, explain how loss to follow-up was addressed<br>Case-control study—If applicable, explain how matching of cases and controls was addressed<br>Cross-sectional study—If applicable, describe analytical methods taking account of sampling strategy	16,21
	12e	Describe any sensitivity analyses	22
**RESULTS**			
Participants	13a	Report numbers of individuals at each stage of study—eg numbers potentially eligible, examined for eligibility, confirmed eligible, included in the study, completing follow-up, and analysed	4
	13b	Give reasons for non-participation at each stage	/
	13c	Consider use of a flow diagram	/
Descriptive Data	14a	Give characteristics of study participants (eg demographic, clinical, social) and information on exposures and potential confounders	5
	14b	Indicate number of participants with missing data for each variable of interest	/
	14c	Cohort study—Summarise follow-up time (eg, average and total amount)	/
Outcome Data	15*	Cohort study—Report numbers of outcome events or summary measures over time<br>Case-control study—Report numbers in each exposure category, or summary measures of exposure<br>Cross-sectional study—Report numbers of outcome events or summary measures	5-8

SECTION	ITEM NUMBER	CHECKLIST ITEM	REPORTED ON PAGE NUMBER:
Main Results	16a	Give unadjusted estimates and, if applicable, confounder-adjusted estimates and their precision (e.g. 95% confidence interval). Make clear which confounders were adjusted for and why they were included	5-8
	16b	Report category boundaries when continuous variables were categorized	/
	16c	If relevant, consider translating estimates of relative risk into absolute risk for a meaningful time period	/
	16d	Report results of any adjustments for multiple comparisons	5-8
Other Analyses	17a	Report other analyses done—e.g. analyses of subgroups and interactions, and sensitivity analyses	/
	17b	If numerous genetic exposures (genetic variants) were examined, summarize results from all analyses undertaken	/
	17c	If detailed results are available elsewhere, state how they can be accessed	/
**DISCUSSION**			
Key Results	18	Summarise key results with reference to study objectives	8
Limitations	19	Discuss limitations of the study, taking into account sources of potential bias or imprecision. Discuss both direction and magnitude of any potential bias	13-15
Interpretation	20	Give a cautious overall interpretation of results considering objectives, limitations, multiplicity of analyses, results from similar studies, and other relevant evidence	8-15
Generalisability	21	Discuss the generalisability (external validity) of the study results<br>Other information	14
**FUNDING**			
	22	Give the source of funding and the role of the funders for the present study and, if applicable, for the original study on which the present article is based	22

*Give information separately for cases and controls in case-control studies and, if applicable, for exposed and unexposed groups in cohort and cross-sectional studies.

49 Spadina Ave. Suite 200
Toronto ON M5V 2J1 Canada
www.biorender.com

Confirmation of Publication and Licensing Rights - Open Access

January 15th, 2026

Subscription Type: *Individual - Academic*
Agreement number: *BD298PFQI2*
Publisher Name: *NPJ parkinson's Disease*

Figure Title: *Figure 3*

Citation to Use: *Created in BioRender. Kaidong, C. (2026) https://BioRender.com/r2uxu7y*

To whom this may concern,

This document ("Confirmation") hereby confirms that Science Suite Inc. dba BioRender ("BioRender") has granted the following BioRender user: Chen Kaidong ("User") a BioRender Academic Publication License in accordance with BioRender's Terms of Service and Academic License Terms ("License Terms") to permit such User to do the following on the condition that all requirements in this Confirmation are met:

1) publish their Completed Graphics created in the BioRender Services containing both User Content and BioRender Content (as both are defined in the License Terms) in publications (journals, textbooks, websites, etc.); and
2) sublicense such Completed Graphics under "open access" publication sublicensing models such as CC-BY 4.0 and more restrictive models, so long as the conditions set forth herein are fully met.

Requirements of User:

1) All Completed Graphics to be published in any publication (journals, textbooks, websites, etc.) must be accompanied by the following citation either as a caption, footnote or reference for each figure that includes a Completed Graphic:
"Created in BioRender. Kaidong, C. (2026) https://BioRender.com/r2uxu7y ".
2) All terms of the License Terms including all Prohibited Uses are fully complied with. E.g. For Academic License Users, no commercial uses (beyond publication in journals, textbooks or websites) are permitted without obtaining or switching to a BioRender Industry Plan.
3) A Reader (defined below) may request that the User allow their figure to be a public template for Readers to view, copy, and modify the figure. It is up to the User to determine what level of access to grant.

Open-Access Journal Readers:

Open-Access journal readers ("Reader") who wish to view and/or re-use a particular Completed Graphic in an Open-Access journal subject to CC-BY sublicensing may do so by clicking on the URL link in the applicable citation for the subject Completed Graphic.

The re-use/modification options below are available after the Reader requests the User to adapt their figure as a BioRender template and the User has granted such access.

1) View-Only/Free Plan Use: A Reader who wishes to only view the Completed Graphic may do so in the BioRender Services as either a BioRender Free Plan user or simply as a viewer. By becoming a BioRender Free Plan user, the Reader may view, modify and re-use the Completed Graphic as permitted under BioRender's Basic License Terms (e.g. personal use only, no publishing or commercial use permitted).
2) Re-Use/Publish with No Modifications: For any re-use and re-publication of a Completed Graphic with no modification(s) to the Completed Graphic made by the Reader, a Reader may do so by citing the original author using the citation noted above with the Completed Graphic. The Reader must also comply with the underlying License Terms which apply to the Completed Graphic as noted above (e.g. no commercial use for Academic License).
3) Re-Use/Publish with Modifications: For any re-use and re-publication of a Completed Graphic with a modification(s) made by the Reader, the Reader may do so by becoming a BioRender user themselves under either an Academic or Industry Plan, citing the original author using the citation noted above with the Completed Graphic and complying with the applicable License Terms.

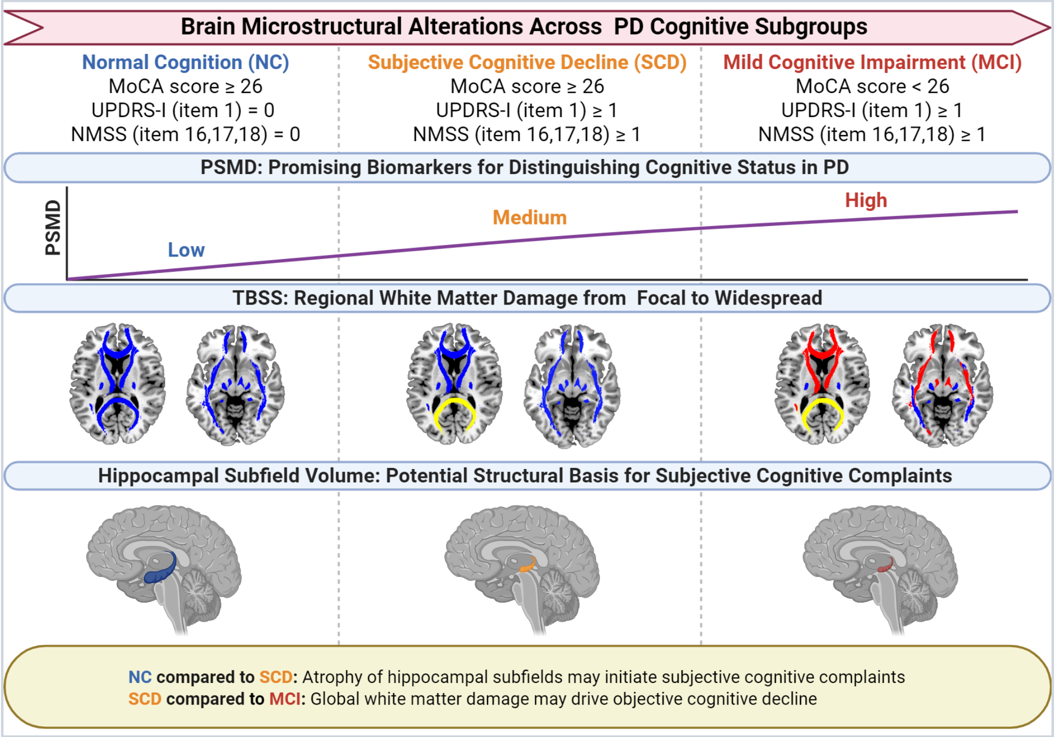

For any questions regarding this document, or other questions about publishing with BioRender, please refer to our BioRender Publication Guide, or contact BioRender Support at support@biorender.com.
